# High-resolution clonal mapping of multi-organ metastasis in triple negative breast cancer

**DOI:** 10.1038/s41467-018-07406-4

**Published:** 2018-11-29

**Authors:** Gloria V. Echeverria, Emily Powell, Sahil Seth, Zhongqi Ge, Alessandro Carugo, Christopher Bristow, Michael Peoples, Frederick Robinson, Huan Qiu, Jiansu Shao, Sabrina L. Jeter-Jones, Xiaomei Zhang, Vandhana Ramamoorthy, Shirong Cai, Wenhui Wu, Giulio Draetta, Stacy L. Moulder, William F. Symmans, Jeffrey T. Chang, Timothy P. Heffernan, Helen Piwnica-Worms

**Affiliations:** 1 Department of Experimental Radiation Oncology, The University of Texas MD Anderson Cancer Center, Houston, 77054 TX USA; 2 Department of Genomic Medicine, The University of Texas MD Anderson Cancer Center, Houston, 77054 TX USA; 3 Institute for Applied Cancer Science, The University of Texas MD Anderson Cancer Center, Houston, 77054 TX USA; 4 Center for Co-Clinical Trials Research, The University of Texas MD Anderson Cancer Center, Houston, 77054 TX USA; 5 Department of Bioinformatics and Computational Biology, The University of Texas MD Anderson Cancer Center, Houston, 77030 TX USA; 6 Department of Integrative Biology and Pharmacology, The University of Texas Health Science Center, Houston, 77030 TX USA; 7 Department of Biostatistics, The University of Texas MD Anderson Cancer Center, Houston, 77030 TX USA; 8 Department of Breast Medical Oncology, The University of Texas MD Anderson Cancer Center, Houston, 77030 TX USA; 9 Department of Pathology, The University of Texas MD Anderson Cancer Center, Houston, 77030 TX USA

## Abstract

Most triple negative breast cancers (TNBCs) are aggressively metastatic with a high degree of intra-tumoral heterogeneity (ITH), but how ITH contributes to metastasis is unclear. Here, clonal dynamics during metastasis were studied in vivo using two patient-derived xenograft (PDX) models established from the treatment-naive primary breast tumors of TNBC patients diagnosed with synchronous metastasis. Genomic sequencing and high-complexity barcode-mediated clonal tracking reveal robust alterations in clonal architecture between primary tumors and corresponding metastases. Polyclonal seeding and maintenance of heterogeneous populations of low-abundance subclones is observed in each metastasis. However, lung, liver, and brain metastases are enriched for an identical population of high-abundance subclones, demonstrating that primary tumor clones harbor properties enabling them to seed and thrive in multiple organ sites. Further, clones that dominate multi-organ metastases share a genomic lineage. Thus, intrinsic properties of rare primary tumor subclones enable the seeding and colonization of metastases in secondary organs in these models.

## Abstract (web summary)

It is unclear how intra-tumoral heterogeneity contributes to metastasis. Here the authors study the clonal dynamics of triple negative breast cancer metastasis using patient derived xenografts and demonstrate that primary tumor clones harbor properties that support seeding and colonization of multiple organs.

## Introduction

Metastasis is the end result of a multi-stage process involving escape of tumor cells from the primary site, survival in circulation, extravasation into foreign organs, and finally, tumor outgrowth at the metastatic site. The ability to complete each stage of metastasis is conferred by inherent genomic attributes of tumor subclones as well as dynamic transcriptomic changes that are influenced by the microenvironment of the primary and metastatic sites^1,2^. TNBC is an aggressively metastatic subtype of breast cancer, with ~35% of patients diagnosed with metastases within five years of initial diagnosis^3^. TNBCs exhibit a broad range of genomic ITH at the time of diagnosis^4–6^, and the level of ITH in the primary tumor is predictive of worse metastasis-free survival in TNBC patients^7^. Interestingly, preservation of minor subclones has been shown to contribute to tumor maintenance^8^.

Genomic analyses comparing primary tumors and metastases in cancer patients have shown that not all primary tumor subclones are equally capable of completing all stages of metastasis, resulting in altered subclonal architecture in metastases relative to their corresponding primary tumors^6,9–25^. However, genomic profiling of patients' tumor samples has several limitations that include limited mutation detection sensitivity due to depth of sequencing limitations and sequencing error rates^26^; imperfect tumor sampling due to spatial ITH; and inability to profile biological replicates, thus diminishing the power to distinguish noise from causality. Thus, genomic profiling does not enable high-resolution mapping of subclonal dynamics during the metastatic process.

With the development of DNA barcoding technologies, it is now possible to trace the lineages of thousands of tumor cell clones in biological replicate mice using next-generation sequencing (NGS)^27^. Barcoding technologies have been employed to track tumor proliferation in vitro^27,28^, tumor cell response to targeted therapies in vitro^29,30^, and tumor growth in vivo^31–34^. A study employing barcoded mouse mammary carcinoma 4T1 cells demonstrated that only a subset of primary tumor subclones entering the bloodstream and surviving as circulating tumor cells had the additional ability to colonize secondary sites^35^. To date, barcoding has not been employed with sufficient resolution to quantify clonal representation of individual lineages in spatially distinct metastatic lesions of human breast tumors during metastasis in vivo.

In this study, a high-complexity library of 50 million unique barcodes was employed to simultaneously label and track the fates of individual tumor cells in vivo in two independent PDX models of treatment-naive TNBC. This high-resolution approach, combined with whole-exome sequencing (WES) and targeted deep sequencing, affords the opportunity to characterize the clonal architecture of primary tumors and their matched metastases in multiple organs. Results reveal reproducible selection of rare populations of primary tumor subclones that become highly abundant in individually isolated metastatic lesions. In addition, lung, liver, and brain metastases share remarkably similar subclonal architecture and are dominated by the same clonal lineages. Genomic sequencing reveals reproducible enrichment of a shared set of mutations in lung, liver, and brain metastases and identifies candidate mutational drivers of metastasis. These data highlight the limitations of bulk primary tumor profiling to capture the biological properties of subclones that contribute to metastatic lesions.

## Results

### Establishment and characterization of a metastatic PDX model

**Fig. 1:**
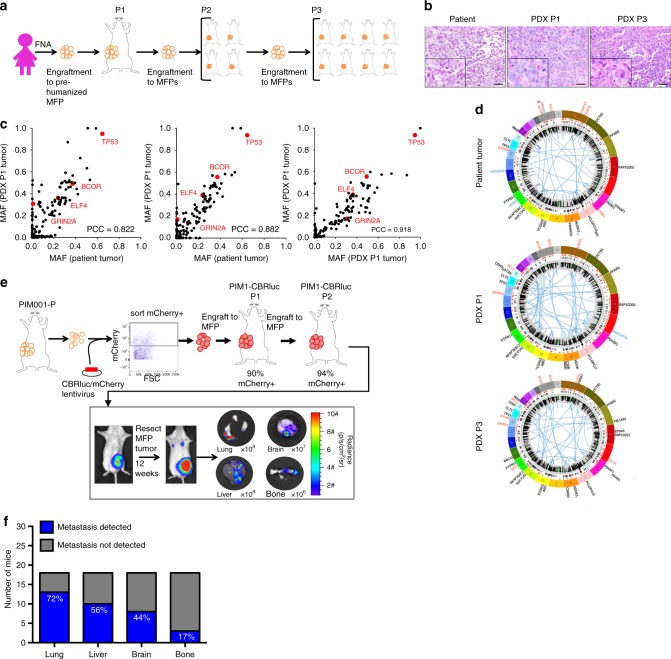
Generation of a genomically heterogeneous PDX model of TNBC. **a** Establishment of the PDX model PIM001-P. FNA, fine-needle aspirate biopsy. MFP, mammary fat pad. **b** Pictomicrographs of H&E-stained sections of the patient’s tumor and PDX tumors are shown. Images are 10×, and insets are 40× magnification. Scale bars represent 100 μm. **c** The mutant allele frequencies (MAFs) of mutations detected by WES and validated by targeted sequencing were compared with each other and with MAFs of the patient’s tumor. Mutations in COSMIC cancer genes are shown in red. Pearson Correlation Coefficients (PCC) are shown in the bottom right corner of each plot. **d** Circos plots were generated to visualize genomic structural rearrangements in patient and PDX tumors. Chromosomes are demarcated by distinct colors. **e** PIM001-P tumor cells were engineered to express click beetle red luciferase (CBRLuc) and mCherry. Following lentiviral transduction, mCherry-positive tumor cells were sorted by FACS and engrafted into the MFP of a NOD/SCID mouse. Tumors were isolated and re-passaged into recipient mice and mCherry positivity was confirmed at each passage by flow cytometry. BLI identified lung, liver, brain, and bone metastases in engrafted mice. In most cases, MFP tumors regrew in the primary site following surgical resection. **f** Percentage of engrafted mice with metastases in indicated organs (*n* = 20)

Tumor cells from the breast of a patient with treatment-naive TNBC, with synchronous metastasis detected at the time of diagnosis, were isolated by fine needle aspiration (FNA) and were engrafted into the pre-humanized mammary fat pads (MFPs) of NOD/SCID mice to generate the PDX model Patient-In-Mouse (PIM)001-P (Fig. 1a). Hematoxylin and eosin staining revealed conservation of histologic features between the patient’s primary tumor biopsy and PDX MFP tumors (Fig. 1b). Specifically, both the patient’s primary tumor biopsy and PDX MFP tumors were characterized by trabecular, pseudopapillary, and solid histologic patterns with abundant tumor vascularity. High magnification microscopy revealed that cancer cells maintained a poorly differentiated appearance with high nuclear-cytoplasmic ratio, prominent nucleoli, and irregular nuclei with vesicular chromatin.

The patient’s primary tumor biopsy, first (P1) and third (P3) passage PDX MFP tumors, and the patient’s blood (germline DNA) were subjected to WES (mean target coverage 183×; Supplementary Data 1). Mutant allele frequencies (MAFs) of non-silent somatic mutations were 80–83% correlated between the patient’s primary tumor biopsy and PDX MFP tumors, and the P1 PDX MFP tumor and the P3 PDX MFP tumor were 95% correlated with one another, demonstrating that the mutational landscape of the PDX tumor was stable during serial passaging in vivo (Supplementary Figure 1a). WES identified 143 somatic non-silent mutations (Supplementary Data 2) with only a few mutations in known cancer genes, including *TP53*_V143fs, *ELF4*_L593H, and *BCOR*_W1475X. Targeted sequencing validation (mean target coverage 1241×) confirmed the high correlation of the patient’s primary tumor biopsy, P1 PDX MFP tumor, and P3 PDX MFP tumor (Fig. 1c and Supplementary Figure 1b). Ten validated low-frequency mutations were detected in PDX MFP tumors (MAF < 0.05) but not in the patient’s primary tumor biopsy and none fell within known cancer genes. Twenty-two validated low-frequency mutations (MAF < 0.13) were unique to the patient’s primary tumor biopsy and none of these fell within known cancer genes. Taken together, these results suggest that rare subclones harboring these mutations were lost upon engraftment or, if maintained in the PDX MFP tumors, were below the threshold of detection by deep sequencing.

Analysis of copy number (CN) alterations and genomic rearrangements in the patient’s primary tumor biopsy and the P1 and P3 PDX MFP tumors revealed widespread CN gains (Fig. 1d and Supplementary Figure 1c), with 25–32% of exonic regions having normal CN status (2N) and 62–70% existing at 3N–6N (Supplementary Data 3). The CN status of genes frequently altered in cancers^36^ was largely concordant between the patient and PDX tumors and included CDK6 which was amplified from 41 copies in the patient’s tumor to 47–50 copies in the PDX MFP tumors (Supplementary Data 4).

### Reproducible subclonal dynamics in lung metastases

**Fig. 2:**
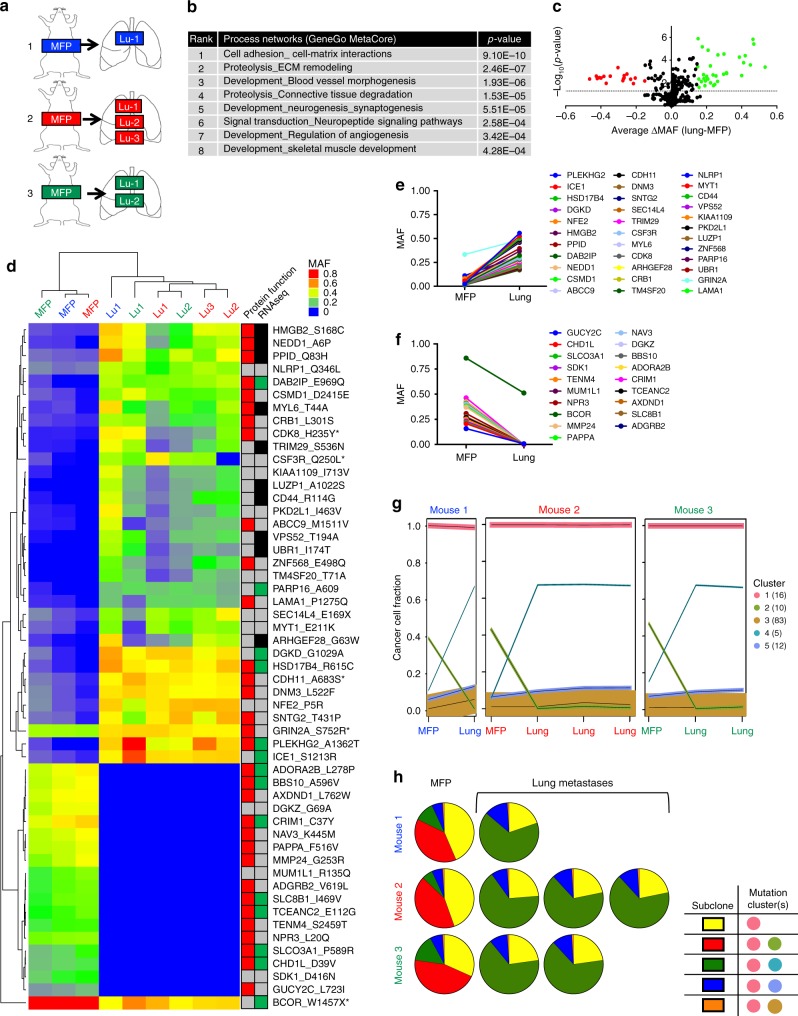
Reproducible selection of genomic subclones in lung metastases. **a** PIM1-CBRLuc primary tumors and lung metastases were isolated from 3 tumor-bearing mice (labeled either blue, red, or green). BLI was used to isolate distinct lung metastases in each mouse. Lu, lung metastasis. **b** RNA isolated from MFP tumors and lung metastases was subjected to RNA-Seq. The top lung metastasis-altered pathways (GeneGo MetaCore) are displayed. **c** Targeted sequencing validation was used to calculate the average change in MAF between lung metastases and MFP tumors. The horizontal dotted line denotes a *p*-value of <0.05. Mutations significantly enriched or depleted in lung metastases by a change in MAF of at least 0.15 are shown in green or red, respectively. **d** Non-silent mutations identified by WES and validated by targeted sequencing significantly enriched or depleted (*p*-value < 0.05, ΔMAF at least 0.15) in lung metastases are shown in the heat map of MAFs calculated from targeted sequencing organized by hierarchical clustering. Predicted amino acid changes are denoted on the right. Mutations predicted to be damaging or potentially damaging to protein function are indicated in red, tolerated or benign mutations are indicated in gray. Mutations detected by RNA-seq are indicated in green, mutant loci covered in RNA-seq without detection of the mutation are indicated in black, and loci without sufficient coverage for assessment of MAF by RNA-seq are indicated in gray. *Mutations falling in COSMIC cancer genes. **e** Average MAFs of validated mutations significantly enriched in lung metastases compared to MFP tumors are plotted. **f** Average MAFs of validated mutations significantly depleted in lung metastases compared to MFP tumors are plotted. **g** The cellular prevalence of mutation clusters as estimated by PyClone are plotted. Line thickness is proportional to the number of mutations within the cluster. The number of mutations comprising each cluster is shown in parentheses. **h** Subclonal architecture was manually reconstructed based on PyClone results. Each pie chart represents a tumor sample, and the colors in the pie chart represent subclones harboring distinct sets of mutation clusters (see **g**)

To enable isolation of spatially distinct metastatic lesions, PIM001-P MFP tumor cells were engineered to express click beetle red luciferase (CBRLuc) and mCherry (PIM1-CBRLuc; Supplementary Figure 1d), then engrafted into the MFPs of recipient mice. Bioluminescence imaging (BLI) of secondary organs ex vivo revealed the presence of tumor cells in physiologically relevant sites including lung, liver, brain, and bone (Fig. 1e, f). MFP tumors and lung metastases shared histological features (Supplementary Figure 1e) and maintained triple negative hormone receptor status (Supplementary Figure 1f). To monitor shifts in clonal architecture during metastasis, primary MFP tumors were resected 12 weeks following engraftment and snap-frozen, and lung metastases were isolated by bioluminescence-guided macro-dissection at mouse necropsy 11–15 weeks later. Genomic DNA (gDNA) and RNA were extracted from metastases, and the relative proportion of human gDNA compared to mouse gDNA was determined by quantitative PCR (Supplementary Figure 2a). The six metastatic lesions with the highest human representation and corresponding MFP tumors from three mice were selected for RNA sequencing (RNA-seq), WES (mean target coverage 209×), and targeted deep sequencing (mean target coverage 1636×; Fig. 2a). MAFs of mutations validated by targeted sequencing were highly concordant with those calculated from WES, with 92% of all MAFs exhibiting <10% variance (Supplementary Figure 2b).

RNA-seq data were computationally purified of mouse sequences. Top pathways significantly altered in PIM1-CBRLuc lung metastases relative to MFP tumors included cell–cell adhesion, proteolysis, and blood vessel morphogenesis (Fig. 2b and Supplementary Data 5). These pathways have well-established roles in human metastasis^37–39^. Importantly, WES and targeted sequencing revealed most mutations were maintained at similar frequencies in PIM1-CBRLuc MFP tumors compared to the patient’s primary tumor biopsy, the P1 parental PDX MFP tumor, and the P3 parental PDX MFP tumor (Supplementary Figure 3). WES and targeted sequencing revealed ITH in primary tumors and metastases (Supplementary Figure 4). Importantly, the spectrum of MAFs was highly correlated between the three replicate PIM1-CBRluc MFP tumors (Supplementary Figure 4b, d), demonstrating that subclonal architecture is a stable characteristic of the PIM1-CBRLuc model, as it is in the parental line (Fig. 1c). Strong shifts in MAFs were observed by WES and targeted sequencing validation of matched MFP tumors and metastases as evidenced by hierarchical clustering, Pearson correlation analysis, and pair-wise distance^40^ (Fig. 2c–f and Supplementary Figure 4). We observed robust, reproducible shifts in validated non-silent MAFs in lung metastases compared to MFP tumors (34 significantly enriched mutations, 19 significantly depleted mutations, average |ΔMAF| ≥ 0.15, *p* ≤ 0.05, Fig. 2c–f, Supplementary Figure 5a, b). Of the 34 validated mutations that were significantly enriched in the lung metastases, 17 were predicted to impact protein function (SIFT^41^ and PolyPhen^42^), and four fell in cancer genes (*CSF3R_Q250L, CDH11_A683S, GRIN2A_S752R*, and *CDK8_H235Y*; Fig. 2d and Supplementary Data 2).

In searching for de novo mutations in lung metastases, we reasoned that we should observe a MAF of 0.0 in all three PIM1-CBRluc MFP tumors by both WES and targeted sequencing, and that a *bona fide* novel mutation would be detected in lung metastases of only a single mouse, as it is highly improbable that the same mutation would arise in independent mice. We detected 14 mutations (identified in WES and validated by targeted sequencing; samples with <7 mutant reads by deep sequencing were considered non-mutant) meeting those criteria (Supplementary Data 6), including: (i) *SCARB2_E54D* at a MAF 0.261 in the single lung metastasis of mouse 1; (ii) *PLEKHG5_S517C*, also detected in a previous study of breast cancer patients^43^, at a MAF 0.324 in only lung metastasis #1 of mouse 2, suggesting the mutation arose after metastatic seeding; (iii) *SLC35C2_L62M*, predicted to be damaging to protein function, at a MAF of 0.184 and 0.147 in the two lung metastases of mouse #3, suggesting that the mutation may have arisen prior to metastatic seeding or that cross-metastasis seeding occurred, and (iv) *CEP78_S17Y*, predicted to be damaging to protein function, at a MAF of 0.448 in only lung metastasis #2 of mouse 3, suggesting the mutation arose after metastatic seeding. Although limitations of detection in NGS prevented definitive determination that these mutations were not present in MFP tumors, these mutations nonetheless mark subclones that became enriched during metastasis and serve as candidate drivers of metastasis.

Rationalizing that mutations capable of functionally driving metastasis would be expressed at the RNA level, we inspected RNA-seq data to detect mutant alleles identified by WES and validated by targeted sequencing. Of the 34 validated mutations significantly enriched in lung metastases (Fig. 2d, e), 15 mutant loci had sufficient RNA-seq coverage to enable assessment of mutations; 6 of these mutations were detected at the RNA level, with all 6 exhibiting MAF enrichment in metastases by both WES and RNA-seq (Supplementary Data 2). Of these 6 mutations, 3 were predicted to impact protein function and included *HSD17B4_R633C, PLEKHG2_A1229T*, and *DAB2IP_E1065Q*. These mutant genes serve as promising candidate drivers of metastasis.

Patterns of CN variations were conserved between PIM1-CBRluc MFP tumors and lung metastases, with the majority of the exome (51-58%) existing at 3N–6N (as estimated from WES data by FACETS^44^ analysis; Supplementary Data 3). Consistent alterations in CN status were observed when comparing lung metastases to MFP tumors, including loss in 5p and 7q and gains in 12q, 19q, 20p, and 22q (Supplementary Figure 5c). These CN alterations could be due to enrichment or depletion of MFP subclones in lung metastases or due to de novo acquisition or loss of CN changes in lung metastases. Interestingly, the consistent CN alterations in lung metastases encompassed several genes whose CNs are frequently altered in cancer^36^ including: gains in *CDK6, BCL2L1, ZNF217, NOTCH1*, and *SMAD4*, (these genes also exhibited increased RNA expression in lung metastases) and losses in *IGF1R, TERT*, and *CDKN2A*.

Next, computational modeling of subclonal architecture was conducted using PyClone^45^, followed by assessment of mutation clustering stability to remove non-robust clusters, then manual reconstruction of subclones (Fig. 2g, h and Supplementary Figure 6). This revealed reproducible shifts in cancer cell frequencies (CCFs) of mutation clusters upon metastasis to the lung (Fig. 2g), with one prevalent MFP subclone lost in all lung metastases and a minor MFP subclone (comprising 6–15% of the population) enriched to predominate the majority of the tumor cell population in each lung metastasis (Fig. 2h). Repeated enrichment of the same mutations in lung metastases and their expression at the RNA level, as well as repeated alterations of CN status in lung metastases, suggest that this selection was based on inherent and/or mutationally driven phenotypes of those subclones that were enriched in lung metastases.

### High complexity barcode-mediated clonal tracking in vivo

**Fig. 3:**
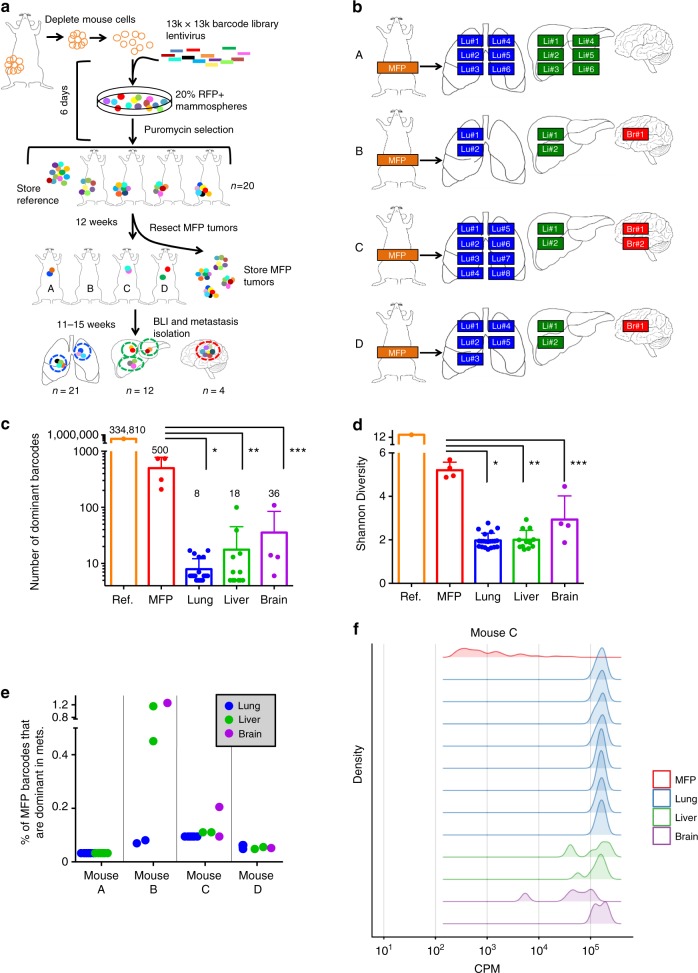
Barcode-mediated clonal tracking of metastasis in vivo. **a** A schematic depiction of the experimental design used for introducing the lentiviral barcode library into freshly isolated PDX tumor cells is shown. Barcoded tumors were engrafted into the MFPs of four recipient mice denoted A, B, C, and D. Due to the high complexity of the barcode library, each mouse was engrafted with cells harboring non-overlapping barcodes. **b** A schematic representation of barcoded MFP tumors and metastatic tumors that were isolated from each mouse for barcode sequencing to monitor clonal dynamics during metastasis. Lu (lung), Li (liver), Br (brain). **c** Unique dominant barcodes (the top 95% most abundant) in each sample were quantified and plotted. The mean quantity of unique barcodes present in each organ site is depicted above each bar. (two-tailed *T*-tests, *p = 6.285e−9, **p = 1.830e−5, ***p = 0.0170; error bars are standard error of the mean, SEM; *n* = 4 MFP tumors, *n* = 21 lung, *n* = 12 liver, and *n* = 4 brain metastases). **d** Shannon Diversity Indices were calculated (in nats: natural digits) taking into account all barcodes for each sample as a measure of ITH. (two-tailed *T*-tests, *p = 9.050e−15, **p = 3.124e−9, ***p = 7.581e−3, error bars are SEM; *n* = 4 MFP tumors, *n* = 21 lung, *n* = 12 liver, and *n* = 4 brain metastases), ref (reference sample). **e** The percent of dominant barcodes (top 95%) in each metastatic lesion relative to the total number of unique barcodes detected in its matched MFP tumor is shown. **f** The distribution (fraction of total barcode reads) of dominant barcodes (top 95%) in each tumor sample from mouse C is shown. CPM (counts per million)

A pooled lentiviral library of greater than 50 million unique DNA barcodes was employed to quantitatively track the lineages of PDX tumor clones during metastasis. To minimize drift due to ex vivo culture, PDX cells were maintained ex vivo for the minimum amount of time possible for lentiviral transduction and antibiotic selection. Cells underwent 1–2 population doublings during this time (Supplementary Figure 7a). A low multiplicity of infection (MOI) was used to ensure that the majority of transduced cells received a single viral integrant (one unique barcode). Due to the high complexity of the library and low MOI, the majority of barcodes present on the day of MFP engraftment were unique to individual tumor cells and were non-redundant between replicate MFP engraftments (Fig. 3a and Supplementary Figure 7b). Thus, comparisons of barcode identity were performed between distinct metastatic lesions within each individual mouse (but not between mice), whereas comparisons of metastasis-induced patterns of clonal dynamics were made both within individual mice and between replicate mice.

To monitor clonal dynamics during metastasis, PIM1-CBRLuc MFP tumor cells were isolated, barcoded, and engrafted into MFPs of mice. MFP tumors were resected and snap-frozen 12 weeks post-engraftment and mice were maintained for an additional 11–15 weeks to allow time for metastatic outgrowth. Spatially distinct metastases in the lung (*n* = 21), liver (*n* = 12), and brain (*n* = 4) were isolated by bioluminescence-guided macro-dissection from four replicate mice (Fig. 3a, b). gDNA and RNA were extracted from each primary and metastasis sample and gDNA was subjected to Illumina NGS. 334,810 unique barcodes were detected in the pre-implantation reference cell pellet (Supplementary Figure 7c), and an average of 14,322 unique barcodes were observed in MFP tumors, corresponding to a tumor-initiating cell (TIC) frequency of 4.28%. The top 95% most abundant barcodes in MFP tumors consisted of an average of 500 unique barcodes. Thus, numerous rare clones were detected in MFP tumors but only 3.49% of clones were abundant in the primary tumor cell population (Fig. 3c). Sequencing of PIM1-CBRluc MFP tumors revealed maintenance of genomic architecture compared to the patient’s primary tumor biopsy and to early-passage PDX MFP tumors (Supplementary Figure 3-4), indicating that the bottleneck observed in barcoded MFP tumors relative to the pre-implantation reference cell pellet was not due to enrichment of a genomic subclone.

### Barcoding reveals clonal selection during metastasis

For subsequent analyses, we defined a clone as the set of cells descended from a single barcoded ancestor, regardless of whether that ancestor represents a genomically and/or phenotypically distinct cell from those harboring different barcodes. Whereas each primary and metastasis sample harbored numerous rare barcodes, only a fraction of these barcodes significantly contributed to the bulk of each sample. Therefore, dominant barcodes, defined as the top 95% most abundant barcodes in each sample, were analyzed (Supplementary Figure 7d). All 37 metastases exhibited reduced Shannon Diversity Indices compared to MFP tumors (Fig. 3d and Supplementary Figure 8a), signifying a reduction of ITH in metastases. Furthermore, there was a significant reduction in the quantity of unique barcodes in all 37 metastases compared to MFP tumors (Fig. 3c and Supplementary Figure 8b), indicating that only a subset of primary tumor clones was significantly enriched in metastases. On average, barcodes dominant in metastases were derived from only 0.03–1.26% (average of 0.14%) of the population of all MFP clones (Fig. 3e).

**Fig. 4:**
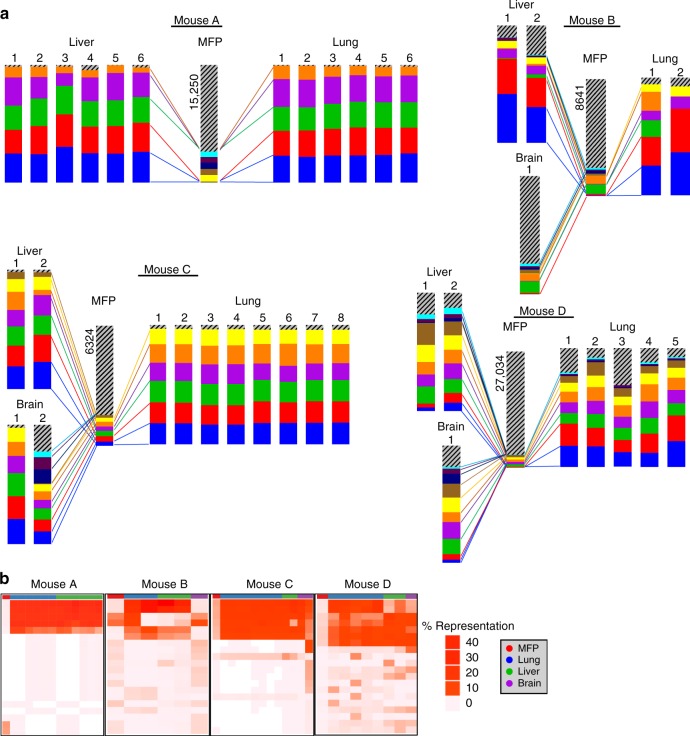
Enrichment of the same barcodes in multiple metastases within each mouse. **a** The frequencies of the 10 most abundant barcodes detected across all samples within each mouse are plotted with different colors for each barcode. Gray striped bars represent all other barcodes in each sample comprising the remainder of the population, and the numbers of these barcodes detected in each MFP tumor is written. Barcodes marked by the same colors in different mice are not identical to one another. Colored lines indicate expansion of primary tumor clones in metastatic lesions, although cross-metastasis seeding may have also occurred. **b** Heat maps showing the representation of the top 20 most abundant barcodes detected across all metastatic samples in each mouse. Undetected barcodes are shown in white

Shifts in clonal architecture were further apparent upon analysis of the overall barcode distribution in each sample, represented by density plots (Fig. 3f and Supplementary Figure 8c). Whereas all four MFP tumors harbored a prominent peak at low barcode abundance (indicating that the bulk of each MFP tumor was comprised of numerous low copy number clones), most metastases exhibited a prominent peak shifted towards higher barcode copy number (indicating that subsets of barcodes were enriched in metastases). However, two metastases exhibited a barcode distribution pattern similar to that observed in their matched MFP tumor (mouse B, liver 1 and 2), despite exhibiting reduced Shannon Diversity and barcode complexity compared to MFP tumors (Supplementary Figure 8a, b). Analysis of individual barcode frequencies in these samples revealed that while a select few barcodes were highly enriched (Fig. 4), some lower frequency barcodes were maintained at a higher frequency than in other metastases. Therefore, these metastases exhibited decreased barcode diversity and complexity compared to mammary tumors while still maintaining a higher-than-average representation of low-frequency barcodes compared with other metastases. Importantly, analysis of the barcodes dominating each mouse’s MFP tumor revealed that the most abundant MFP barcodes did not typically dominate corresponding metastases (Supplementary Figure 9), and conversely that the dominant metastatic barcodes in each mouse were present at low frequencies in the matched MFP tumor (Fig. 4). Concordantly, low Jaccard overlap indices were observed when comparing each MFP tumor against its metastases (Supplementary Figure 10). These results demonstrate robust and reproducible shifts in clonal architecture in metastases of various organ sites in this PDX model. Furthermore, metastases were predominantly derived from minor primary tumor clones in agreement with the pattern of subclonal dynamics observed in WES analysis of lung metastases (Fig. 2d–g).

### Overlapping clones dominate metastases in diverse organs

Due to the high complexity of the barcode library, direct comparison of barcode identity between mice was not possible. However, within each mouse all metastases were derived from the same pool of MFP tumor barcodes, allowing direct comparisons of barcode identity between spatially distinct metastatic lesions. Analysis of barcode distributions revealed strong shifts in clonal architecture in metastases (Fig. 3f and Supplementary Figure 8c). Within each mouse, comparison of dominant clones in individual metastatic lesions isolated from the lung, liver, and brain revealed that the majority of dominant barcodes in each metastatic lesion was shared with other metastases in that mouse (Fig. 4). Strikingly, 36 of 37 lung, liver, and brain metastases were dominated by very few barcodes (≤10 in each mouse) and these barcodes were identical across all metastases within each mouse (Fig. 4), suggesting that non-random selection had occurred. The widespread overlap of dominant clones across lung, liver, and brain metastases in each mouse indicates that these subclones lack intrinsic organotropism for establishment and survival in metastatic sites. One exception to this pattern was the brain metastasis in mouse B where we observed a similar pattern of clonal architecture as was observed in the corresponding MFP tumor (Fig. 4). This could indicate that the metastasis seeded late and was harvested at an earlier stage, and thus a subset of barcodes had not yet had the time to dominate the metastatic population. The finding that the vast majority of metastases were dominated by the same barcodes, most of which were represented at a similar frequency in each metastasis in each mouse, suggested that a tumor-cell intrinsic property provided a selective advantage to these clones enabling them to dominate multiple metastatic sites.

### Numerous clones seed, but few dominate, metastases

**Fig. 5:**
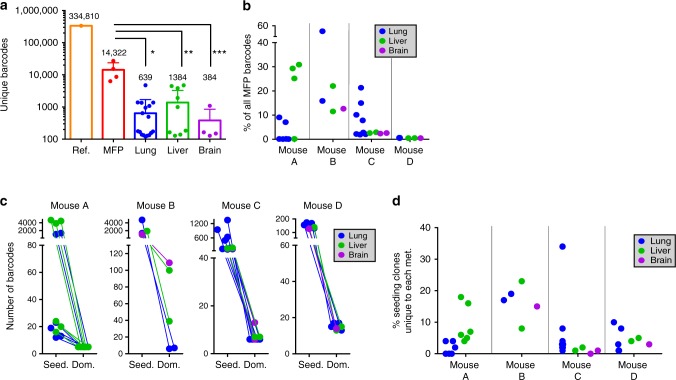
Metastases harbor numerous low-abundance seeding clones. **a** Number of seeding clones, defined as barcodes detected regardless of copy number, is plotted. Barcodes present in metastases were only counted if they were also detected in the corresponding MFP tumor. The mean number of barcodes in each sample is denoted above each bar. (two-tailed *T*-tests, *p = 2.65e−7, **p = 0.00025, ***p = 0.024; error bars are SEM; *n* = 4 MFP tumors, *n* = 21 lung, *n* = 12 liver, and *n* = 4 brain metastases). **b** The percent of seeding clones detected in each metastatic lesion relative to the total number of unique barcodes detected in its matched MFP tumor is shown. **c** The number of dominant barcodes in metastases of each organ was compared with the total number of seeding clones detected in each metastasis. **d** The percentage of seeding clones that are unique to each metastatic site (detected in the MFP tumor of that mouse, but not in any other metastasis of that mouse) is plotted

Given the level of variability between technical replicates, expected sequencing error rates, and enhanced detection power for rare barcodes in metastases due to the fact they are much smaller than MFP tumors, we reasoned that detection of any barcode in the MFP tumor, in addition to the metastasis, was not a false-positive (Supplementary Figures 6c–d and 8). We thus defined seeding clones as any clonal lineage detected in both the MFP and at least one metastasis in a mouse. The total number of seeding barcodes, regardless of their abundance, and their copy numbers in each metastatic site were quantified. Metastasis-seeding clones were derived from a median of 2.37% of all MFP tumor barcodes (Fig. 5a, b; whereas the majority of metastases were seeded by a minority population from the MFP, several samples were outliers and skewed the distribution towards an average of 8.03%). In the majority of metastases, a striking difference was observed in the number of unique dominant clones and the number of unique seeding clones (metastases harbored 10–1000-fold more seeding clones than dominant clones; Fig. 5c). 7 out of 12 metastases in mouse A harbored a number of seeding clones that was only two to four times higher than the number of dominant clones (Fig. 5c, left panel). However, each of these seven metastases also harbored several thousand rare unique barcodes that were not detected in the MFP (likely due to differences in limit of detection between metastases and MFP tumors), indicating that numerous rare lineages seeded these seven independent metastatic lesions. The majority of seeding clones detected in each metastasis were detected in at least one other metastatic lesion in that mouse, suggesting that seeding events may be non-stochastic (Fig. 5d). The marked discrepancy between the number of seeding and dominant barcodes in the majority of metastases indicated that while many clones had the capacity to seed metastatic sites, only a select few clones were endowed with the capacity to dominate metastases.

### Functional analysis of metastasis-initiating frequency

Limiting dilution transplantation assays were performed on tumor cells from two PDX models to determine metastasis-initiating frequencies in a functional assay (Table 1). BLI was used to quantitate the frequency of metastases in mice that grew MFP tumors. Transplantation of as few as 10 PIM1-CBRLuc cells yielded metastases in some mice, with a lung metastasis-initiating frequency of 17.9%, whereas the lung metastasis-initiating frequency of BC3_A2 was calculated to be 0.7%. These results demonstrate that only a subset of tumor clones was capable of completing all stages of the metastatic cascade, confirming results obtained with barcoding and WES. Furthermore, the finding that transplantation of only 10 PIM1-CBRluc cells into the MFP could yield metastases to multiple organ sites in some mice supports the lack of organotropism observed by barcode-mediated clonal tracking.

**Table 1:** Limiting dilution transplantation to measure metastasis-initiating frequency

	Cell number	Lung	Liver	Brain
PIM1-CBRluc	100	4/4 (100%)	1/3 (33%)	1/3 (33%)
PIM1-CBRluc	10	5/6 (83%)	2/3 (67%)	1/3 (33%)
PIM1-CBRluc	M.I.F.	17.9% (6.6–48.5%)	2.1% (0.6–7.6%)	0.7% (0.2–3.3%)
BC3_A2	100	3/5 (60%)	NT	NT
BC3_A2	10	0/8 (0%)	NT	NT
BC3_A2	M.I.F.	0.7% (0.2–2.4%)	N/A	N/A

Of mice that grew MFP tumors upon transplantation of tumor cells in limiting dilutions, the frequency of metastasis was quantified with BLI ex vivo in two PDX models. The number of organs that harbored metastases is shown over the number of organs assayed by BLI, and the percent of positive organs is shown in parentheses. The estimated metastasis-initiating frequency (MIF, 95% confidence intervals shown in parentheses) is shown for each site

NT: not tested, N/A: not applicable

### Clonal selection during lung metastasis in a second PDX

**Fig. 6:**
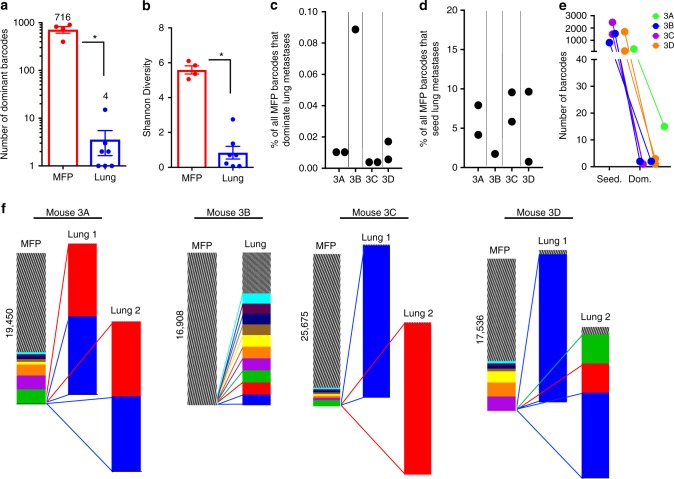
An additional PDX model reveals clonal selection during metastasis. **a** In BC3_A2, unique dominant barcodes in each sample were quantified and plotted. The mean quantity of unique barcodes present in each site is depicted above each bar. (two-tailed *T*-test, *p = 9.40e−6; error bars are SEM; *n* = 4 MFP tumors, *n* = 7 lung metastases). **b** In BC3_A2, Shannon Diversity Indices were calculated (in nats: natural digits) taking into account all barcodes for each sample as a measure of ITH. (two-tailed *T*-test, *p = 7.55e−6; error bars are SEM; *n* = 4 MFP tumors, *n* = 7 lung metastases). **c** In BC3_A2, the percent of dominant barcodes (top 95%) in each metastatic lesion relative to the total number of unique barcodes detected in its matched MFP tumor is shown (labeled 3A–3D). **d** In BC3_A2, the percent of seeding clones detected in each metastatic lesion relative to the total number of unique barcodes detected in its matched MFP tumor is shown. **e** In BC3_A2, the number of dominant barcodes in metastases of each organ was compared with the total number of seeding clones detected in each metastasis. **f** In BC3_A2, the frequencies of the 10 most abundant barcodes detected across all samples within each mouse are plotted in different colors. Gray striped bars represent all other barcodes in each sample comprising the remainder of the population, and the numbers of these barcodes detected in each MFP tumor is written. Barcodes marked by the same colors in different mice are not identical to one another. Colored lines indicate expansion of primary tumor clones in metastatic lesions, although cross-metastasis seeding may have also occurred

Barcode mediated clonal tracking was performed in a second CBRluc-labeled PDX model of TNBC (BC3_A2^46,47^), for which molecular^47^ and metastatic^46^ features were previously characterized, to determine if clonal selection is a common feature of metastasis in these models. This PDX model was derived from the primary tumor of a treatment-naive TNBC patient that was diagnosed with synchronous metastasis^47^. Due to the relatively low metastasis-initiating frequency of BC3_A2 (Table 1), it was only possible to assess several lung metastases in this model. Consistent with results obtained with PIM1-CBRluc, each metastasis was comprised of thousands of heterogeneous, low abundance barcodes and few dominant (<15) barcodes (Fig. 6a–d and Supplementary Figure 11). The magnitude of the clonal bottleneck in lung metastases from BC3_A2 tumors was greater than in PIM1-CBRLuc, with some lung metastases dominated by only 1–3 barcodes (Fig. 6a and Supplementary Figure 11a, b). The average lung metastasis-initiating frequency in BC3_A2 tumors was 5.66% (Fig. 6d), but only 0.02% of MFP clones became dominant in each metastasis (Fig. 6c). Consistent with observations made in PIM1-CBRluc, identical clones dominated distinct lung metastases in two of the three BC3_A2 mice bearing multiple lung metastases (mouse 3A and 3D). The third mouse (3C) had two lung metastases that were each dominated by a single, distinct barcode (Fig. 6f). It is yet unclear if these two barcodes mark unique clones or identical phenotypic/genotypic clones. Taken together, results from two independent PDX models revealed that numerous low abundance clones were capable of seeding metastases, but only a select few became highly enriched and could do so in spatially distinct metastases.

### Shared molecular features in multi-organ metastases

**Fig. 7:**
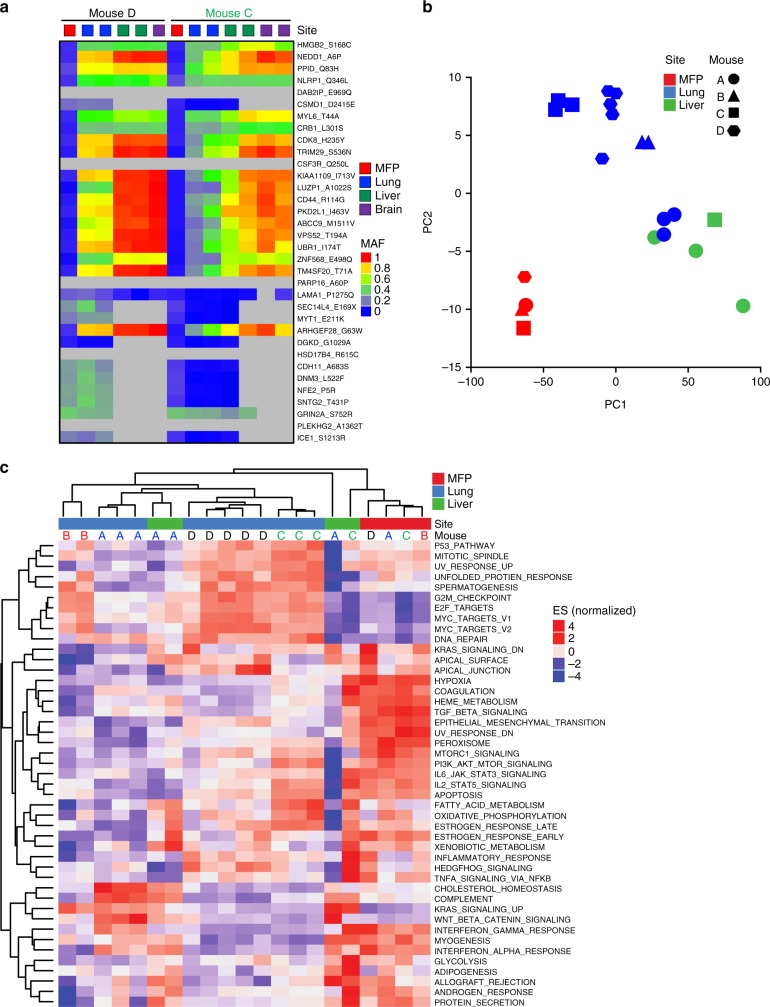
Genomic and transcriptomic characterization of metastases. **a** Of the 34 mutations found to be significantly enriched in PIM1-CBRLuc lung metastases (Fig. 2d; mutations are displayed in the same order in this figure), MAFs were calculated using targeted deep sequencing data generated from two barcoded mice harboring MFP tumors and lung, liver, and brain metastases. A heatmap of MAFs is shown. Grayed cells indicate insufficient coverage at that genomic region in that sample for accurate assessment of MAF. **b** Principal component analysis (PCA) of RNA-seq data was conducted. **c** GSVA pathways are shown in a heatmap of normalized enrichment scores organized by unsupervised hierarchical clustering

To determine if the genomic subclones enriched in lung metastases (Fig. 2) were also enriched in liver and/or brain metastases, we conducted targeted deep sequencing of those genomic regions mutated in PIM001-P using barcoded PIM1-CBRLuc MFP tumors and lung, liver, and brain metastases. While high levels of contaminating mouse stromal cells due to small lesion size led to relatively low coverage for some human genes in liver and brain samples, we were able to determine MAFs of those mutations for which there was sufficient coverage (see methods, Supplementary Data 1 and 8). Of the 34 mutations enriched in lung metastases (Fig. 2c–e), 29 had sufficient coverage in at least one metastatic lesion to enable calculation of MAF (Fig. 7a). Of those, 18 mutations were highly enriched in all three organ sites compared to their matched MFP tumor (Fig. 7a). These data provide compelling evidence that lung, liver, and brain metastases were derived from a shared genomic lineage and identify 18 potential driver mutations of multi-organ metastasis.

RNA-seq analysis of a subset of barcoded lung metastases, liver metastases, and MFP tumors revealed that metastases harbored distinct transcriptomes from their corresponding MFP tumors. Principal component analysis revealed that the transcriptomes of lung and liver metastases clustered more closely to each other than to MFP tumors, and metastases clustered according to organ site (Fig. 7b and Supplementary Data 7). Interestingly, unsupervised hierarchical clustering of gene set enrichment patterns and gene expression levels revealed that some liver metastases clustered with lung metastases and separately from MFP tumors (Fig. 7c and Supplementary Figure 12). While the activation status of some pathways was concordant between liver and lung metastases (such as downregulation of EMT, TGF-β signaling, and hypoxia in metastases compared to MFP tumors), some pathways were differentially regulated in the two sites (e.g., cholesterol homeostasis). Together, these results demonstrate that while the clonal and genomic origins of metastases in distinct organs are similar, gene expression programs can be more variable, likely due to the diverse organ microenvironments.

## Discussion

In this study, barcoding was employed to map primary tumor lineages in multiple metastatic sites in vivo with clone-level resolution. The PDX models used in this study were derived from the primary breast tumors of untreated TNBC patients with concurrent metastases at the time of diagnosis and thus represent aggressive disease. An advantage of this experimental design is the capacity to measure the clonal dynamics of metastasis to multiple organ sites in replicate mice without therapy-induced selection. Importantly, the observed concordance of metastases in diverse secondary organs is a feature of human breast cancer metastasis^48–50^.

Genomic sequencing studies have been performed on metastases isolated from patients who received therapy prior to metastatic sampling and thus are not directly comparable to our study. These studies revealed enrichment of primary tumor subclones, evolution of new mutations, and shifts in copy number status in metastases relative to primary tumors^6,13,17–19,21–25^. Recent studies of matched breast tumors and metastases revealed that metastases typically derived from a subclonal population of breast tumor cells and that the majority of mutations detected in metastases were also present in the breast tumor^22,23,25,49,50^. While these studies encompass the evolution of tumor cells as they metastasize and undergo treatment, studies in our PDX models reflect the natural history of metastasis in the absence of the selective pressure of therapy. Despite limitations of PDX models, namely, that they require the use of immune-compromised mice to host human tumors and that their establishment can inadvertently enrich for aggressive clones^51^, we observed shifts in clonal architecture during metastasis as have been reported in patient’s metastases^11,12,22–24,48,50^. Furthermore, our findings are in agreement with a recent study that profiled nine primary TNBCs matched with multi-organ metastases obtained from a rapid autopsy program^49^. This study found that metastases were polyclonal, that metastases in diverse secondary organs shared genomic subclones and gene expression patterns within each patient, and that the majority of mutations found in metastases pre-existed in the primary tumor. Although patients in this study received interventional therapies prior to metastatic sampling, the findings are consistent with those from our PDX models.

We obtained direct evidence for the existence of thousands of seeding clones in metastases in both PDX models. Due to the high complexity of the barcode library and the negative-binomial distribution of barcodes in the reference cell pellet, it is highly unlikely that the thousands of MFP clones marked by unique barcodes were all phenotypically identical. At a minimum, it is clear that numerous tumor cells marked by unique barcodes seeded each metastasis and were maintained at a low frequency. While monoclonal seeding of metastases has been reported in some cancer patients^50,52^, our findings are in agreement with recent genomic studies demonstrating polyclonal seeding of metastases^6,12,22,23,25,49^.

Although we cannot rule out the possibility that multiple barcodes marked the same genomic and/or phenotypic clone(s), dominance of identical barcodes across secondary organs within each mouse suggests that tumor cell-intrinsic properties contributed to the preferential outgrowth of a select subpopulation in three organ microenvironments. Human TNBC frequently spreads to multiple distal organs within an individual patient^53^ (most often the lung, liver, bone, and brain^54,55^), suggesting that primary tumor clones have the capacity to colonize and thrive in multiple organ microenvironments. The overlap in dominant barcodes between distinct metastases could be explained by: (1) these subclones seeded each individual metastasis in individual metastatic events, (2) these subclones were the earliest to escape the MFP and thus had a selective advantage to dominate each metastasis, or (3) these subclones seeded and dominated one metastatic lesion then secondarily seeded other metastases. Horizontal cross-seeding of metastases has been observed in human breast cancer^50^. Regardless of the route taken by each subclone, it is clear that these subclones harbor properties enabling them to seed and thrive in diverse organ microenvironments. Future lineage tracing studies to monitor serially obtained metastases from PDX models will elucidate the temporal nature of the clonal dynamics underlying metastasis.

Our data suggest that sampling of primary tumors may not effectively capture the biological features of metastases and that therapies targeting metastatic subpopulations may effectively target metastases in multiple organ sites. Future analyses of clonal lineages across multiple organ sites in PDX models derived from additional patients will be critical to determine the generalizability of these patterns. Further, the evolution of single-cell sequencing and imaging technologies will afford the opportunity to investigate mechanisms enabling rare MFP tumor cell populations to colonize secondary organs and will inform strategies to preclude the preferential outgrowth of these clones in multiple organ sites.

## Methods

### Study approval

This study was carried out in accordance with the recommendations in the Guide for the Care and Use of Laboratory Animals from the National Institutes of Health (NIH) Institutional Animal Care and Use Committee (IACUC). The protocol was approved by the IACUC at MD Anderson Cancer Center. Mice were euthanized when they became moribund or when they reached defined study end points. Animals were euthanized as dictated by the Association for Assessment and Accreditation of Laboratory Animal Care International and IACUC euthanasia endpoints. Informed consent was obtained from all human participants, and all relevant ethical regulations were followed as approved by the Institutional Review Board (IRB) at MD Anderson. Patient biopsies were obtained through an approved IRB protocol at the University of Texas MD Anderson Cancer Center (protocol 2011-0007).

### PIM001-P PDX model generation

The PDX model PIM001-P was established^56^ following a modified published protocol^57^. Briefly, the fourth MFPs of 3-week to 5-week old NOD/SCID mice (NOD.CB17-Prkdc^scid^/NcrCrl, Charles River, NCI Colony) were pre-humanized with GFP-labeled immortalized human mammary stromal fibroblasts (EG fibroblasts, derived from a reduction mammoplasty and immortalized with telomerase reverse transcriptase, TERT, and engineered to express green fluorescent protein, GFP^57^) a minimum of 3–4 weeks prior to tumor cell implantation. A FNA was obtained from the breast tumor of a patient diagnosed with metastatic TNBC who had not yet received any therapeutic intervention. Tumor cells were kept on ice following biopsy and transferred to the laboratory within 0.5–1 h. Tumor cells were pelleted by centrifugation at 1200×*g*, washed with DME:F12 supplemented with 5% bovine calf serum (BCS), and resuspended in red blood cell (RBC) lysis buffer (Sigma R7757). Cells were pelleted by centrifugation, resuspended in DME:F12 with 5% BCS, and filtered through a 70–100 μm sterile filter. Cell number and viability was quantified by Acridine Orange/Propidium Iodine (AO/PI) staining on a fluorescence-coupled CellOMeter Nexcelom (61% viability). Tumor cells were mixed with EG fibroblasts and 1/3 volume Matrigel (Corning) and injected into pre-humanized MFPs of recipient mice. Cells in Matrigel were maintained on ice until engraftment. 6.5 × 10^4^ viable patient biopsy tumor cells and 3 × 10^4^ EG fibroblasts were implanted to the pre-humanized MFPs of two independent mice (one tumor per mouse).

When tumors reached approximately 1000 mm^3^, they were harvested and dissociated into single cells and organoids by mechanical mincing followed by digestion with 3 mg mL^−1^ collagenase (Roche) and 0.6 mg mL^−1^ hyaluronidase (Sigma) supplemented with 1.3% bovine serum albumin (BSA) (Sigma) in DME:F12 media containing antibiotics. Tumor digests were incubated on a rotating platform for 2–4 h at 37 °C. Digested tumor cells were processed as described above for patient FNA samples. One million viable tumor cells were resuspended in 50% volume Matrigel and injected into MFPs of NOD/SCID mice that had been pre-humanized with one million human EG fibroblasts (50% irradiated with 4 Gy + 50% non-irradiated)^57^. At each passage, aliquots of dissociated tumor cells were stored in cryopreservation media in order to maintain a frozen stock of PIM001-P tumor cells.

### Quality control analyses of PDX tumor samples

An aliquot of digested tumor cells from P1 and P3 were pelleted, and gDNA was extracted using the DNeasy Blood & Tissue kit (Qiagen). The relative quantity of human and mouse tumor cells was assessed with qPCR using gDNA as a template. Human gDNA was assayed using a TaqMan probe and primer set that specifically recognized the human *RNaseP* gene (20× human *RNaseP* copy number assay, FAM-TAMRA, Life Technologies). Mouse gDNA was assayed using a TaqMan probe and primer set that specifically recognized the mouse *Trfc* gene (20× mouse *Trfc* copy number assay, VIC-TAMRA, Life Technologies). gDNA from purely human and mouse cell lines were used as absolute calibrators for each species. qPCR was conducted using the 2× TaqMan gene expression master mix (Life Technologies). The ΔΔ*C*_t_ method was used to calculate the relative ratio of human and mouse gDNA in each tumor sample. The percent of human DNA was calculated as:

% Human genomic DNA = 100 × 2^-Δ*C*t,human^ * (2^-Δ*C*t,human + ^2^-Δ*C*t,mouse^)^-1^(1)

To assess whether the co-implanted human EG fibroblasts (stably expressing GFP) were cleared after tumor formation, gDNA was subjected to PCR against the gene encoding *GFP* using 50 ng of template DNA with TEMPase 2× hot start polymerase (Apex) according to the manufacturer’s specifications. Amplicons were visualized by agarose gel electrophoresis and ethidium bromide staining to detect *GFP* amplicons. PCR using primers recognizing the gene encoding *GAPDH* was used as a positive control to ensure the quantity and quality of DNA was sufficient for PCR analysis. Primer pairs for GFP were: Fwd 5′-AAGTTCATCTGCACCACCG; Rev 5′-TCCTTGAAGAAGATGGTGCG. Primer pairs for GFP were: Fwd 5′-ACATCATCCCTGCCTCTAC; Rev 5′-TCAAAGGTGGAGGAGTGG.

Short-tandem repeat (STR) DNA fingerprinting was performed on gDNA from P1 and P3 tumors to establish and verify the identity of the PDX line and to verify that the PDX line was not cross-contaminated with cells from another origin. gDNA (50 ng) from P1 and P3 tumors was submitted to the MDACC Characterized Cell Line Core (CCLC, Cancer Center Support Grant-funded NCI # CA016672) for STR profiling. The Promega 16 High Sensitivity STR Kit (Catalog # DC2100) was used for fingerprinting analysis, and the STR profiles were compared to online search databases (DSMZ/ ATCC/ JCRB/ RIKEN) of approximately 2500 known profiles, as well as the MDACC CCLC database of approximately 2600 known DNA fingerprint profiles. Each PDX model had a unique STR profile, un-matched with any profiles in the checked databases, and P1 and P3 tumor samples had matching profiles.

### Histologic evaluation of patient and PDX samples

Patient and PDX tumor biopsies stored as formalin-fixed paraffin embedded tissue blocks were cut into 5 μm sections and analyzed by hematoxylin and eosin (H & E) staining. Histologic properties were evaluated by a breast pathologist (Dr. F. Symmans) Immunohistochemical staining was performed using standard methods. Briefly, after deparaffinization of the sections with xylene and rehydration via graded aqueous ethanol baths, antigen retrieval was performed using Reveal decloaker solution (Biocare Medical, RV1000 M) and an EX-Retriever v.3.0 microwave. Endogenous peroxidases were quenched by treatment with Dako Dual Enzyme block (S2003). The Estrogen Receptor antibody (Neomarkers MS-750-S, culture supernatant) was diluted 1:50; the Progesterone Receptor antibody (Neomarkers RB-9017-p1) was diluted 1:50 (0.2 mg mL^−1^); the HER2 antibody (Thermo Fisher Scientific PA5-14632) was diluted 1:300. Antigen-antibody complexes were tagged with ImmPRESS reagent (Vector Laboratories). The M.O.M kit was used for ER staining (Vector Laboratories MP-2400); ImmPRESS Reagent Anti-Rabbit IgG kit was used for PR and HER2 staining (Vector Laboratories MP-7401).

### Generation of a labeled sub-line of PIM001-P

To generate a sub-line of PIM001-P expressing fluorescent and bioluminescent markers, a PIM001-P tumor was harvested at P4 and dissociated into single cells. Following mouse cell depletion by magnetic-activated cell sorting according to the manufacturer’s protocol (mouse cell depletion cocktail, Miltenyi), 2 million human tumor cells were plated in mammosphere conditions using 5 mL of complete Mammocult medium (StemCell Technologies) in ultra-low attachment plates (Corning) in the presence of lentivirus (approximate MOI = 8) encoding Click beetle red luciferase (CBRLuc) and mCherry. Twelve hours later, polybrene (Sigma) was added at a concentration of 10 µg mL^-1^. Twenty-four hours after addition of virus, the media was refreshed. Cells were cultured at 5% CO_2_. Four days after lentiviral transduction, the cell population (containing single cells and organoids) was pelleted, organoids were dissociated with TrypLE Express dissociation reagent (Gibco), washed, and resuspended in PBS containing 0.5% BSA. Cells were stained with Sytox Blue viability dye (Life Technologies), then run through a BD Aria Fusion fluorescence activated cell sorter (FACS). Of all viable cells in the population, 35% were mCherry positive. The viable mCherry-positive fraction was collected and an aliquot was analyzed on the FACS sorter in a post-sort purity check, which confirmed that the sorted population contained no mCherry-negative cells. 50,000 viable mCherry-positive tumor cells were washed and engrafted into the MFP of one NOD/SCID mouse as described above in the presence of EG fibroblasts. Half of the EG fibroblasts were irradiated with 4 Gy and mixed with non-irradiated fibroblasts^46^. Once the tumor reached 1000 mm^3^, it was harvested, dissociated into single cells and immediately re-transplanted into 9 recipient mice (one million cells each). This process was repeated on second-passage CBRLuc labeled tumor cells. Aliquots of cells were stored in cryopreservation media and frozen after each passage.

Using first-passage and second-passage PIM1-CBRLuc tumor cell aliquots (P1 and P2 after introduction of the markers), we confirmed the human identity of the PDX tumors by flow cytometry analysis of freshly dissociated tumor cells stained with an antibody against mouse-specific MHC class I (anti-H2kd clone SF1-1.1, BioLegend catalog number 116601, used at a dilution of 1:1000). Maintenance of the bioluminescent and fluorescent markers was confirmed by flow cytometry analysis for mCherry. Clearance of human EG fibroblasts from PIM1-CBRLuc tumors was confirmed by flow cytometric detection of GFP. To confirm that PIM1-CBRLuc tumors were maintained as a pure PDX sub-line free of contamination with other known human cell sources, we conducted STR profiling of PIM1-CBRLuc tumors and found that at each passage, their STR profile matched that of the parental PIM001-P PDX line.

### Isolating metastases from PDX tumor-bearing mice

Prior to euthanasia, mice were subjected to whole body BLI. BLI was performed following published procedures^46,58^. Briefly, animals were administered an intraperitoneal injection of d-luciferin (150 μg g^-1^ body weight; GoldBio) in phosphate-buffered saline. Ten minutes after injection, isoflurane-anesthetized animals were imaged with a charge-coupled device camera-based BLI system (IVIS Lumina and IVIS Spectrum; PerkinElmer). Signals were displayed as photons/s/cm^2^ per sr for image representation. Living Image Software was used to manually define regions of interest, and quantified data were expressed as total photon flux (photons/s). To quantify organ distribution, d-luciferin was administered to live animals as described above, and tissues were assessed with BLI ex vivo at necropsy (~4 months following tumor engraftment). Lungs, livers, and brains were subjected to BLI individually. BC3_A2 metastases to liver and brain were not observed under the conditions used for this study, possibly because MFP tumors were resected in a survival surgery to minimize barcode loss to necrosis. Bioluminescent images were used to macro-dissect metastatic lesions. Each spatially distinct metastatic lesion was dissected using isopropanol-sterilized tools and was snap-frozen individually. Organs exhibiting regions of bioluminescence with Gaussian distribution were counted as one nodule under 10 s acquisition times to calculate frequency of metastasis to individual organs.

For the WES metastasis experiment, qPCR was conducted on MFP tumors and lung metastases to quantify the relative proportion of human gDNA present in the mouse background. In order to be confident in mutant allele frequencies calculated from WES data, we selected the 3 replicate mice harboring lung metastases with the greatest amount of human representation to obtain high depth of sequencing coverage. qPCR to quantify human and mouse gDNA was conducted as described above.

### Limiting dilution transplantation assays

PIM1-CBRLuc and BC3_A2 cells were processed and implanted as described above following serial dilution to the indicated cell numbers. PIM1-CBRLuc (passage 9) cells were engrafted in the presence of 50% Matrigel (Corning), then tumors were resected in a survival surgery when they reached approximately 1.0 cm in diameter. Mice were maintained for an additional 12-16 weeks, then euthanized. Lungs, livers, and brains were resected and subjected to BLI ex vivo as described above.

BC3_A2 cells (derived from the treatment-naive primary tumor biopsy of a TNBC patient, propagated as a PDX^47^, then engineered to have knockdown of *TP53*^46^) were mixed with human EG fibroblasts (50% irradiated with 4 Gy mixed with 50% non-irradiated; 50% tumor cell number) and serially diluted. The indicated cell numbers were implanted to mouse MFPs as described above. When tumors reached maximum burden (2 cm diameter) or mice reached moribund endpoints, lungs were subjected to BLI ex vivo to assess metastasis. Organs exhibiting regions of bioluminescence with Gaussian distribution were counted as positive with a 10 s exposure time and smallest binning. The lung metastasis-initiating frequency was calculated in mice bearing MFP tumors whose lungs were assayed by BLI^59^.

### Barcoding PIM1-CBRLuc and BC3_A2 PDX tumor cells

We first confirmed whether PIM1-CBRLuc cells would maintain viability ex vivo for the time required for viral transduction and antibiotic selection. Following mouse cell depletion, viable human tumor cells were plated in mammosphere conditions (MammoCult, StemCell Technologies) in ultra-low-attachment 96-well plates (Corning) at a density of 1000 cells per well. Viability was monitored by Cell-Titer-Glo (Promega) luminescence assays according to the manufacturer’s protocol and imaged using a ClarioStar luminescence plate reader (BMC Lab Tech). We observed that cells maintained viability for approximately 2 weeks following tumor digestion, but that viability began decreasing after 6 days in mammosphere conditions. Cells did not grow under 2D culture conditions. As detailed below, a brief selection (48 h) in puromycin was necessary to kill non-barcoded cells prior to engraftment. We used an MOI of 0.2 to ensure that the vast majority of transduced cells received only one barcode each. Thus, at this low MOI, ~80% of the tumor cell population would not contain a barcode, thus prohibiting the capacity to accurately quantify clonal architecture and make comparisons between each sample. For this reason, freshly isolated PDX tumor cells were immediately transduced with the barcode library, then selected with puromycin and re-introduced into mice in the shortest amount of time possible to allow for lentiviral expression and killing of non-transduced cells by puromycin (6 days after tumor isolation and infection). Details of this protocol are provided below.

PIM1-CBRLuc tumors were harvested, pooled, dissociated into single cells, and depleted of mouse stroma by magnetic-activated cell sorting according to the manufacturer’s protocol (Miltenyi, mouse cell depletion cocktail). Depletion of mouse cells was validated by flow cytometry analysis of cells stained with an antibody against mouse-specific MHC class I (anti-H2kd clone SF1-1.1, BioLegend catalog number 116101, used at a dilution of 1:1000). For the metastasis experiment, following mouse cell depletion, 60 million viable human tumor cells were plated in mammosphere conditions (MammoCult, StemCell Technologies) in ultra-low-attachment plates (Corning) at a density of 1.25 × 10^6^ cells mL^−1^. Immediately following plating, the pooled lentiviral barcode library was added to cells (Cellecta, CellTracker 50 M packaged lentiviral barcode library, Cat. # BC13X13-30M-V) at an MOI of 0.2 to ensure that each infected cell received only one single barcode, including 10 µg mL^-1^ polybrene (Sigma-Aldrich). Cells were maintained at 5% CO_**2**_. Media was replaced with fresh MammoCult 24 h following addition of lentivirus. Cells were maintained in mammosphere conditions for an additional 48 h. At that time (36 h following tumor digestion), media was refreshed and puromycin was added to a final concentration of 3 µg mL^–1^. An aliquot of non-transduced cells was maintained and treated with puromycin to confirm complete cell killing. Cells were maintained in puromycin-containing media for a total of 36 h. An aliquot of cells was used to confirm appropriate transduction efficiency by flow cytometry to detect RFP (present in the lentiviral backbone) on day 5 following lentiviral transduction.

A total of 72 h following tumor cell digestion, cells were pelleted, washed, resuspended in fresh MammoCult, and counted. One million viable barcoded cells in MammoCult were combined with 50% Matrigel (Corning) and engrafted into the non-pre-humanized MFPs of NOD/SCID mice (*n* = 3 PIM001-P tumors for the complexity test and *n* = 20 PIM1-CBRLuc tumors for the metastasis experiment; these two experiments were conducted separately and thus two separate lentiviral transductions were conducted on these two PDX lines). Two pre-implantation reference pellets of one million cells each were snap-frozen at that time.

For metastasis experiments (both the WES cohort and the barcoded cohort), PIM1-CBRLuc mammary tumors were monitored until they reached ~1000 mm^3^ in order to allow for the most robust metastatic seeding as possible while avoiding excessive necrosis. MFP tumors were resected in a survival surgery and total tumors were snap-frozen. Mice were monitored for an additional 11–15 weeks until declining health was observed. This allowed the maximum amount of time possible for detectable metastatic lesions to grow. Metastases were harvested as described above. One mouse employed in the barcoding study (mouse A) had a thymic lymphoma at study end point. However, it is important to note that the patterns of clonal architecture were similar in this mouse compared with its biological replicates (that did not have thymic lymphomas), demonstrating that the presence of the lymphoma in this one mouse did not affect clonal dynamics. The rate of overt thymic lymphomas in NOD/SCID mice in our colony is 25% (calculated from a total of 159 mice at greater than ~6 months of age).

BC3_A2 cells were originally derived from the tumor of a patient with treatment-naive TNBC^46,47,60^. Cells were cultured in DMEM + 10% FBS and maintained at 37 °C, 5 % CO_**2**_, and 5% O_2_ in humidified air. Cells were transduced with lentiviral vectors to silence expression of endogenous wild type p53 and to express CBRLuc and mCherry and selected with hygromycin (shRNA against p53) and zeocin (CBRLuc/ mCherry). Cells were then transduced with the barcode library (MOI 0.2) in the presence of 1 μg mL^−1^ polybrene and incubated under 2D culture conditions. Two days after transduction, puromycin was added at a final concentration of 1 μg mL^−1^. Four days later (6 days after lentiviral infection), transduced cells were implanted into the fourth MFPs of recipient mice in the presence of EG fibroblasts (50% irradiated with 4 Gy + 50% non-irradiated). 0.92  million tumor cells and 0.45^6^ million EG cells were implanted to each MFP in 1/3 volume Matrigel. Tumors were removed when they reached 8–10 mm in diameter. Mice were euthanized approximately 6 months post-engraftment, and tissues were isolated and processed as described for PIM1-CBRLuc.

### Nucleic acid extraction

RNA and DNA were both extracted from each PDX tumor sample that had been harvested by snap-freezing tumor pieces. For non-barcoded tumor cells, total tumors were homogenized in Buffer RLT containing beta-mercaptoethanol (Qiagen) using a Bullet Blender (Next Advance) and navy bead lysis kit. Homogenates were then processed using an RNeasy kit (Qiagen). RNA was DNase-treated (Turbo DNase Kit, Ambion), then purified and concentrated using an RNA cleanup kit (Zymo Research) according to the manufacturer’s instructions. For DNA extraction, flow-through from the RNeasy columns from the first two RNeasy kit steps were collected, combined, and precipitated with an equal volume of 100% ethanol at −20 °C for at least 2 h. After pelleting the precipitated DNA, the pellet was resuspended in buffer ATL containing proteinase K (Qiagen) and samples were extracted using the DNeasy kit (Qiagen).

For barcoded tumor cells, tissues and RNA were prepared as described above. For DNA extraction, the flow-through from the RNeasy extraction was mixed with equal volume of Phenol:Chloroform:Isoamyl Alcohol (25:24:1 pH8.0, Sigma Aldrich). After centrifugation (13,500×*g*, 12 min), the upper phase was transferred to a new tube and a second extraction step with Chloroform:Isoamyl Alcohol (24:1, Sigma Aldrich) was performed. Again, the upper phase was transferred to a new tube and added with 0.1 volumes of 3 M NaOAc (Sigma Aldrich) and 0.8 volumes of Isopropanol (Fisher Scientific) to precipitate gDNA. Samples from in vitro cells were stored over-night at −20 °C before centrifugation. Centrifugation of tumor samples was performed at 18,400×*g* for 20 min. The DNA pellet was washed once in 70% Ethanol (Fisher Scientific) and centrifuged again for 5 min at 18,400×*g*. The DNA pellet was air-dried for 5–10 min and dissolved over-night in UltraPure distilled water at room temperature (Thermo Fisher). The final DNA concentration was assessed by NanoDrop 2000 (Thermo Scientific) quantification.

### Sequencing patient samples and early-passage PDX tumors

The germline sample for the patient PIM001, PIM001-P patient tumor samples, PIM001-P-P1 PDX, and PIM001-P-P3 PDX were analyzed by WES at the Cancer Genetics Laboratory at MD Anderson Cancer Center. Genomic libraries from approximately 500 ng gDNA were prepared using the standard KAPA paired-end sample preparation kit (KAPA Biosystems) according to the manufacturer’s instructions. SureSelect Human All Exon Kit, version 4 (Agilent Technologies) was used to enrich sequencing libraries for exomes. Samples were pooled 2 per lane, and paired-end 2 × 75 bp sequencing was performed using the Illumina HiSeq 2000.

### Whole-exome sequencing of PIM1-CBRLuc tumors

PIM1-CBRLuc primary MFP tumors and lung metastases were analyzed by WES performed by the Sequencing and Microarray Facility at MD Anderson Cancer Center. Libraries were prepared from 200 ng of Biorupter ultrasonicator (Diagenode)-sheared gDNA using the Agilent SureSelectXT Reagent Kit (Agilent Technologies). Libraries were prepared for capture with ten cycles of PCR amplification, then assessed for size distribution on a Fragment Analyzer using the High Sensitivity NGS Fragment Analysis Kit (Advanced Analyticals) and quantified using the Qubit dsDNA HS Assay Kit (ThermoFisher). Exon target capture was performed using the Agilent SureSelectXT Human All Exon V4 kit. Following capture, index tags were added to the exon enriched libraries using six cycles of PCR. The indexed libraries were then assessed for size distribution using the Agilent TapeStation and quantified using the Qubit dsDNA HS Assay Kit. Libraries were sequenced either one library per lane (5 samples) or equal molar concentrations of 3 libraries were pooled and sequenced in two lanes of the HiSeq4000 sequencer, using the 75nt paired end format.

### Whole-exome sequencing data analysis

We processed the NGS data using the BETSY expert system^61^. Reads from contaminating mouse sequences were subtracted from the PDX sequencing data. We discarded adapter and low-quality sequences using Trimmomatic and aligned the trimmed reads against the GRCm38 mouse genome assembly using the BWA aligner. We discarded all reads that aligned with up to one mismatch. We aligned the remaining non-mouse reads to the human reference (hg19) following the GATK best practices. Bam files were sorted, and indexed using samtools. Duplicates were identified using Picard tools and indels were realigned using GATK. To identify somatic variants, we employed a consensus approach^62,63^ consisting of seven algorithms to call SNPs, short, and long indels (MuSE, MuTect, MuTect2, Pindel, Radia, SomaticSniper, Strelka, Varscan2, and mutationSeq). For somatic variant callers, we used the patient’s blood sample as the germline reference. For the Strelka analysis, we skipped the depth filter, and set minPruning = 3 in MuTect2 to speed up computation. We annotated the variants using Annovar and SnpEff. Silent variants, as well as those falling into intergenic and intronic regions, were removed. Somatic variants were further screened and variants meeting these criteria were eliminated from all downstream analyses: identified by only 1 variant caller, coverage (reference allele + alternate allele) less than 30 in all samples, alternate allele coverage less than 5 in all samples, germline VAF≥1%, and presence in the 1000 Genomes Project database. We also applied a custom filter designed to identify alignment artifacts from repeat regions^64^. These manifest as a high density of mutations that cannot be confirmed by other techniques, such as Sanger sequencing. To do this, we performed pairwise comparisons for the presence of pairs of mutations seen in the same read. We scored the correlation between each pair using a Chi-square test and flagged each one that is significant with false discovery rate < 5%. Of those, we discarded any that were correlated in over 1% of the reads. All remaining mutations were manually checked and suspected false positives, as determined by consistent positioning near the ends of reads, consistent strand specificity, poor mapping quality, and consistent co-occurrence with other alternate alleles on the same read +/−50 base pairs, were removed.

The change in MAF between MFP tumors and lung metastases was calculated as: ΔMAF = average MAF across all lung metastases—average MAF across all MFP tumors. The p-value associated with each ΔMAF was calculated using two-sample two-tailed *t*-tests. Pearson Correlation Coefficients were calculated to evaluate the relatedness of mutations between each pair of samples. Hierarchical clustering using Euclidean distance and complete linkage generated heat-maps of MAFs. Amino acid change annotations in heatmaps were generated by ANNOVAR.

### Custom targeted panel deep DNA sequencing and analysis

Mutations detected by WES meeting the filtering criteria described above were selected for validation by targeted deep DNA sequencing. In collaboration with Admera Health, LLC (South Plainfield, New Jersey), custom probe design was performed on SureDesign Custom Design Tool (Agilent Technologies) to target 629 SNPs across the human genome. Final probe design included low to moderate stringency to cover all desired regions. Repeat regions with RepeatMasker and WindowsMasker were confirmed by uploading the AllTracks.bed in UCSC genome browser against hg19 genome and checking the overlap between missed regions and the UCSC RepeatMasker track. For that reason, missed regions were reduced to low stringency setting.

Libraries were prepared with starting input of 100–1000 ng of genomic DNA and was sheared using Covaris S220 system (Covaris). Libraries were pooled into seven separate pools and targets were captured by hybridization using the SureSelect XT capture method. Quality and quantity checks were done on a TapeStation D1000 High Sensitivity and Qubit 2.0 dsDNA HS assay (Life Technologies). Average library bp size was observed at approximately 400 bp. Sequencing was completed on the Nextseq 500/550 System High Output 150 Cycles to target 2000× mean coverage. Adapters were trimmed on BaseSpace utilizing the fastq toolkit.

To call variants from the targeted sequencing reads, we started by removing the reads that aligned to the mouse genome as described above. We aligned and pre-processed the reads using the same procedure, leaving out the mark duplicates step. We called somatic variants against the same germline sample profiled by whole exome sequencing. To filter mutations in the targeted sequencing data, of mutations detected by WES targeted in the panel, we selected mutations called by at least 2 of the mutation callers in at least one sample in the targeted sequencing data. Next, we required at least 3 mutant reads to be detected, otherwise the mutation was considered not detected.

For targeted sequencing of two barcoded mice (MFP tumors, lung metastases, liver metastases, and brain metastases), we implemented the above cutoffs, but increased the minimum total coverage to 100 reads, due to the generally low depth of coverage achieved in liver and brain metastases due to high levels of mouse stroma contamination.

### Copy number alteration analysis

Analysis of copy number alteration for paired tumor-normal whole exome sequencing samples was performed using *FACETS*^44^. To detect major changes, critical value for segmentation was set to 300, and minimum number of heterozygous SNPs in a segment used for bivariate t-statistic during clustering was set to 30. To reduce hyper-segmentation, scanning window size was set to 500 bp and 250 bp for lung metastasis and patient-PDX sets separately. Other parameters remain the same as default values. From the segmentation files produced by FACETS, we extracted the estimated total copy number (under column tcn.em) and plotted the values from 1000 evenly spaced coordinates across the human genome (from chromosomes 1 to 22) as a heatmap.

### Computational modeling of clonal architecture

To select variants for subclone analysis, we discarded those with low (<35) or high (>1000) read depth in any samples, low allele frequency in all samples (<1%), high germline allele frequency (>1%), and those that appeared to be errors by manual inspection of the alignments. We discarded variants with high total copy number (>6), and also those that occurred in regions where the copy number varied across samples. This resulted in 138 variants common to all nine samples. For the FACETS analysis of mouse 2’s MFP sample, we followed the same procedure as above, except we used nbhd = 200 to maintain a consistent ploidy (~3) across all samples. All samples were predicted to have high purity (0.94–0.98).

Using the read count, copy number, and purity prediction for the selected variants, we used PyClone^45^ to estimate the CCFs. We used the run_analysis_pipeline script in Pyclone (v0.13) under the default settings: base_measure_params (alpha = 1; beta = 1), beta_binomial_precision_params (prior rate = 0.001; shape = 1; proposal precision = 0.01; value = 1000), concentration (prior rate = 0.001; shape = 1; value = 1), density = pyclone_ beta_binomial, num_iters = 10,000, and error_rates = 0.001 for all samples. Output statistics and plots were generated after 2500 samples of burn-in.

PyClone analysis identified 11 clusters comprising one or more variants. We noticed that some clusters were unstable, and were comprised of variants that were frequently assigned to other clusters. Therefore, we assessed the robustness of each cluster. To this end, we first calculated a pairwise matrix containing the probabilities that each pair of variants were assigned to the same cluster. We discretized these probabilities into a low category (0.5 ≤ *p* < 0.9) and a high category (0.9 ≤ *p* ≤ 1). Then, we calculated whether the frequency of these categories deviated from random using a Chi-square test. We accepted a cluster as robust if the distribution of probabilities significantly deviated from random, accepting *p* < 0.05 after a Bonferroni correction. This resulted in five stable mutation clusters (126 variants in total).

### RNA sequencing

For non-barcoded samples, strand-specific total RNA sequencing was conducted by the MDACC Sequencing and Microarray Facility using RNA extracted from non-barcoded PIM1-CBRLuc mammary tumors and lung metastases (the same samples which had been analyzed by WES). Barcoded, Illumina compatible stranded total RNA libraries were prepared using the TruSeq Stranded Total RNA Sample Preparation Kit (Illumina). Briefly 250 ng of DNase I treated total RNA was depleted of cytoplasmic and mitochondrial ribosomal RNA (rRNA) using Ribo-Zero Gold (Illumina). After purification, the RNA was fragmented using divalent cations and double stranded cDNA was synthesized using random primers. The ends of the resulting double stranded cDNA fragments were repaired, 5′-phosphorylated, 3′-A tailed and Illumina-specific indexed adapters were ligated. The products were purified and enriched with 12 cycles of PCR to create the final cDNA library. The libraries were quantified using the Qubit dsDNA HS Assay Kit and assessed for size distribution using the Agilent TapeStation (Agilent Technologies), then multiplexed 9 libraries per pool (exceptions: mouse 2 lung metastasis 1 and 2 were each sequenced in their own lane). Library pools were quantified by qPCR and sequenced in two lanes of the Illumina HiSeq4000 sequencer using the 75 bp paired end format.

RNA sequencing of barcoded mammary tumors, lung, and liver metastases was conducted by Admera Health (Plainfield, NJ). Isolated RNA quality was assessed by Bioanalyzer 2100 Eukaryote Total RNA Nano (Agilent Technologies). Libraries were constructed with KAPA RNA HyperPrep with Riboerase and performed based on manufacturer’s recommendations. Final library quantity was measured by KAPA SYBR® FAST qPCR and library quality evaluated by Tapestation RNA HS Assay (Agilent Technologies). Equimolar pooling of libraries was performed based on qPCR values and loaded onto Illumina Hiseq platform.

### RNA sequencing data analysis

For non-barcoded samples, we discarded the contaminating mouse reads from the RNA-seq data using the same procedure as above, except aligning with the STAR aligner against the GRCm38 transcriptome using a GTF from GENCODE. We estimated expression as TPM values using RSEM, and counts using HTSeq-Count. Gene expression analysis was conducted after subtraction of mouse reads using DEseq2. For variant calling, we used a similar pipeline as for the WES analysis, except including a Split’N’Trim step. Significantly differentially expressed genes in lung metastases compared to mammary tumors were defined as those with a log_2_(fold change) of at least 2.0 and false-discovery rate of ≤0.05. Pathways of genes significantly differentially expressed in lung metastases were identified using GeneGo MetaCore (Thomson Reuters). RNA-seq data was mined to analyze structural variants using MapSplice and TopHat fusion. Structural variants are illustrated in circos plots.

For barcoded samples, Fastq files were aligned to human (gencode v19) and mouse transcriptome (m11) simultaneously using kallisto [v0.43] to enable dissecting mouse and human RNA into separate bins. Gene level counts were then derived by summing transcript counts of all human protein coding genes, and used for downstream analysis. Samples with low numbers of reads which formed separate clusters in principal component analysis and other QC metrics were removed. This included brain metastases from all mice and several liver samples. Genes expressed in all samples were used for single-sample GSEA and gene level clustering analysis. Fifty pathways from MSigDB hallmark set was used as a reference for ssGSEA, performed using implementation by Bioconductor package GSVA.

### Barcode amplification and sequencing

For NGS library generation, the barcodes were amplified starting from the total amount of genomic DNA in 2 rounds of PCR using the Titanium Taq DNA polymerase (Clontech-Takara) and pooling the total material from the first PCR before proceeding with the second round. The first PCR reactions were performed for 16 cycles with 13K_R2 (5′-AGTAGCGTGAAGAGCAGAGAA-3′) and FHTS3 (5′-TCGGATTCAAGCAAAAGACGGCATA-3′). The second PCR reactions were performed with P7_NF16-13 × 13 (5′-CAAGCAGAAGACGGCATACGAGATGCAGAAGACGGCATACGAAGACAGTTCG-3′) and P5-IND## (5′- ACGGCGACCACCGAGATCTACACGCACGACGAGACGCAGACGAANNNNNNAGAGAACGAGCACCGACAACAACGCAGA-3′). It was critical to amplify relatively the same amount of product from PCR1, so volumes of PCR1 and cycles of PCR2 were adjusted to normalize PCR2 products per batch. Primers for the second PCR reactions were optimized to introduce the required adapters for Illumina NGS technology. PCR amplification products were analyzed by agarose gel electrophoresis (2.5%, Lonza) for the expected 279 bp size. Amplified PCR products from 2 replicates of the second PCR reactions were pooled and extracted from agarose gel with the QIAquick gel purification kit (Qiagen). Library samples were sent to Admera Health (South Plainfield, NJ) for NGS on an Illumina NextSeq500 with Fseq16IND (5′- TCTGCGTTGTTGTCGGTGCTCGTTCTCT-3′) and Rseq16BC (5′-AGCTCGAGGTTCAGAGTTCTACAGTCCGAA-3′) and Rseq16IND (5′ ACACGCACGACGAGACGCAGACGAA-3′) as sequencing primers. Two sets of quality control replicates were performed to evaluate the consistency of PCR based library generation. First, PCR2 conditions were repeated using the same PCR1 products of a select set of metastasis samples on two different library preparation days. Secondly, using a single MFP tumor, we performed PCR2 from 1/16, 1/4, and total pooled PCR1 volumes. These QC libraries were then submitted along with the experimental samples for NGS.

### Barcode data processing

A custom analysis pipeline was used to quantify barcodes from FASTQ files. Reads were first filtered based on the presence of a 4 bp spacer (TTCG) between the two barcodes, positions 19–22 bp. Reads which had the spacer, within a hamming distance of 1 bp, were used for downstream analysis.

The reads were then split into two separate fastq files (1–18 bp, 23–40 bp), and both barcodes (BC1 and BC2) were aligned to the 13 K library, preserving their pairing. Bowtie (v2.2.3) was used to perform the alignment allowing 1 bp mismatch at either end of the barcode. These parameters were optimized to enable maximal alignment, while minimizing alignment of reads to multiple barcodes. Further, reads where both barcodes aligned to the library uniquely were preserved for counting. We used SAMtools to extract the reads and perform counting of paired reads^65^.

### Barcode data analysis and normalization

Counts obtained using methods described above were normalized for library size, by calculating counts per million for each barcode (i):

2 $${\mathrm{CPM}}_i = \frac{{{\mathrm{Counts}}_i}}{{{\sum} {{\mathrm{Counts}}} }}10^6$$

This enables comparisons of barcodes within and across samples. We performed further filtering of the barcodes by optimizing the overlap between technical replicates from the MFP of mouse B (Supplementary Figure 12). By calculating a cumulative sum of CPM, we extracted barcodes representing the top 90, 95, and 99% of the tumor mass. We used the top 95% barcodes of each sample for subsequent analysis for density curves, Jaccard indices, and all analyses of dominant barcodes. To further understand the dynamics of barcode distribution, we calculated the maximum CPM achieved by a barcode across all tumors of a specific mouse (Supplementary Figure 13), which revealed that the majority of barcodes remain at low numbers.

### Code availability

Code used in this study is available from the authors upon written request.

## Electronic supplementary material

Supplementary Information

Description of Additional Supplementary Files

Supplementary Data 1

Supplementary Data 2

Supplementary Data 3

Supplementary Data 4

Supplementary Data 5

Supplementary Data 6

Supplementary Data 7

Supplementary Data 8

Reporting Summary

## Data Availability

Whole-exome sequencing, targeted DNA sequencing, and RNA sequencing data generated in this study are available from dbGAP with accession code phs001742.v1.p1 (Figures 1c-d, 2b-h, 7, Supplementary Figures 1a-c, 2b, 4, 5, 6, and 12). Pre-processed gene expression profiles have been deposited in the NCBI Gene Expression Omnibus with accession code GSE120753 (Figs. 2b, 7b, c, and Supplementary Figure 12). A reporting summary for this Article is available as a Supplementary Information file.

## References

[1] Fidler IJ (2001). Seed and soil revisited: contribution of the organ microenvironment to cancer metastasis. Surg. Oncol. Clin. N. Am..

[2] Chambers AF, Groom AC, MacDonald IC (2002). Dissemination and growth of cancer cells in metastatic sites. Nat. Rev. Cancer.

[3] Pogoda K, Niwinska A, Murawska M, Pienkowski T (2013). Analysis of pattern, time and risk factors influencing recurrence in triple-negative breast cancer patients. Med. Oncol..

[4] Nik-Zainal S (2012). The life history of 21 breast cancers. Cell.

[5] Shah SP (2012). The clonal and mutational evolution spectrum of primary triple-negative breast cancers. Nature.

[6] Yates LR (2015). Subclonal diversification of primary breast cancer revealed by multiregion sequencing. Nat. Med..

[7] Yang F (2017). Intratumor heterogeneity predicts metastasis of triple-negative breast cancer. Carcinogenesis.

[8] Marusyk A (2014). Non-cell-autonomous driving of tumour growth supports sub-clonal heterogeneity. Nature.

[9] Schwarz RF (2015). Spatial and temporal heterogeneity in high-grade serous ovarian cancer: a phylogenetic analysis. PLoS Med..

[10] de Bruin EC (2014). Spatial and temporal diversity in genomic instability processes defines lung cancer evolution. Science.

[11] Gerlinger M (2012). Intratumor heterogeneity and branched evolution revealed by multiregion sequencing. N. Engl. J. Med..

[12] Gundem G (2015). The evolutionary history of lethal metastatic prostate cancer. Nature.

[13] Ding L (2014). Clonal architectures and driver mutations in metastatic melanomas. PLoS One.

[14] Campbell PJ (2010). The patterns and dynamics of genomic instability in metastatic pancreatic cancer. Nature.

[15] Xie T (2014). Patterns of somatic alterations between matched primary and metastatic colorectal tumors characterized by whole-genome sequencing. Genomics.

[16] Yachida S (2010). Distant metastasis occurs late during the genetic evolution of pancreatic cancer. Nature.

[17] Brastianos PK (2015). Genomic characterization of brain metastases reveals branched evolution and potential therapeutic targets. Cancer Discov..

[18] Moelans CB (2014). Genomic evolution from primary breast carcinoma to distant metastasis: few copy number changes of breast cancer related genes. Cancer Lett..

[19] Meric-Bernstam F (2014). Concordance of genomic alterations between primary and recurrent breast cancer. Mol. Cancer Ther..

[20] Ding L (2010). Genome remodelling in a basal-like breast cancer metastasis and xenograft. Nature.

[21] Navin N (2011). Tumour evolution inferred by single-cell sequencing. Nature.

[22] Savas P (2016). The subclonal architecture of metastatic breast cancer: results from a prospective community-based rapid autopsy Program “CASCADE”. PLoS Med..

[23] Hoadley KA (2016). Tumor evolution in two patients with basal-like breast cancer: a retrospective genomics study of multiple metastases. PLoS Med..

[24] Liu W (2009). Copy number analysis indicates monoclonal origin of lethal metastatic prostate cancer. Nat. Med..

[25] Yates LR (2017). Genomic evolution of breast cancer metastasis and relapse. Cancer Cell.

[26] Robasky K, Lewis NE, Church GM (2014). The role of replicates for error mitigation in next-generation sequencing. Nat. Rev. Genet..

[27] Nolan-Stevaux O (2013). Measurement of cancer cell growth heterogeneity through lentiviral barcoding identifies clonal dominance as a characteristic of in vivo tumor engraftment. PLoS One.

[28] Pal D (2016). Long-term in vitro maintenance of clonal abundance and leukaemia-initiating potential in acute lymphoblastic leukaemia. Leukemia.

[29] Hata AN (2016). Tumor cells can follow distinct evolutionary paths to become resistant to epidermal growth factor receptor inhibition. Nat. Med..

[30] Bhang HE (2015). Studying clonal dynamics in response to cancer therapy using high-complexity barcoding. Nat. Med..

[31] Nguyen, L. V. et al. DNA barcoding reveals diverse growth kinetics of human breast tumour subclones in serially passaged xenografts. *Nat. Commun*. **5**, 5871 (2014). 10.1038/ncomms6871 PMC4284657 25532760

[32] Nguyen LV (2014). Clonal analysis via barcoding reveals diverse growth and differentiation of transplanted mouse and human mammary stem cells. Cell Stem Cell.

[33] Belderbos ME (2017). Clonal selection and asymmetric distribution of human leukemia in murine xenografts revealed by cellular barcoding. Blood.

[34] Klauke K (2015). Tracing dynamics and clonal heterogeneity of Cbx7-induced leukemic stem cells by cellular barcoding. Stem Cell Rep..

[35] Wagenblast E (2015). A model of breast cancer heterogeneity reveals vascular mimicry as a driver of metastasis. Nature.

[36] Zack TI (2013). Pan-cancer patterns of somatic copy number alteration. Nat. Genet..

[37] Pezzella F, Harris AL, Tavassoli M, Gatter KC (2015). Blood vessels and cancer much more than just angiogenesis. Cell Death Discov..

[38] Wolf K, Friedl P (2011). Extracellular matrix determinants of proteolytic and non-proteolytic cell migration. Trends Cell Biol..

[39] Ludwig T (2005). Local proteolytic activity in tumor cell invasion and metastasis. Bioessays.

[40] Shibata D (2011). Mutation and epigenetic molecular clocks in cancer. Carcinogenesis.

[41] Kumar P, Henikoff S, Ng PC (2009). Predicting the effects of coding non-synonymous variants on protein function using the SIFT algorithm. Nat. Protoc..

[42] Adzhubei Ivan, Jordan Daniel M., Sunyaev Shamil R. (2013). Predicting Functional Effect of Human Missense Mutations Using PolyPhen-2. Current Protocols in Human Genetics.

[43] Tommiska J (2006). Evaluation of RAD50 in familial breast cancer predisposition. Int. J. Cancer.

[44] Shen R, Seshan VE (2016). FACETS: allele-specific copy number and clonal heterogeneity analysis tool for high-throughput DNA sequencing. Nucleic Acids Res..

[45] Roth A (2014). PyClone: statistical inference of clonal population structure in cancer. Nat. Methods.

[46] Powell E (2016). p53 deficiency linked to B cell translocation gene 2 (BTG2) loss enhances metastatic potential by promoting tumor growth in primary and metastatic sites in patient-derived xenograft (PDX) models of triple-negative breast cancer. Breast Cancer Res..

[47] Li S (2013). Endocrine-therapy-resistant ESR1 variants revealed by genomic characterization of breast-cancer-derived xenografts. Cell Rep..

[48] Almendro V (2014). Genetic and phenotypic diversity in breast tumor metastases. Cancer Res..

[49] Siegel MB (2018). Integrated RNA and DNA sequencing reveals early drivers of metastatic breast cancer. J. Clin. Invest..

[50] Brown D (2017). Phylogenetic analysis of metastatic progression in breast cancer using somatic mutations and copy number aberrations. Nat. Commun..

[51] Eirew P (2015). Dynamics of genomic clones in breast cancer patient xenografts at single-cell resolution. Nature.

[52] McPherson A (2016). Divergent modes of clonal spread and intraperitoneal mixing in high-grade serous ovarian cancer. Nat. Genet..

[53] Tseng LM (2013). Distant metastasis in triple-negative breast cancer. Neoplasma.

[54] Savci-Heijink CD (2015). Retrospective analysis of metastatic behaviour of breast cancer subtypes. Breast Cancer Res. Treat..

[55] Foulkes WD, Smith IE, Reis-Filho JS (2010). Triple-negative breast cancer. N. Engl. J. Med..

[56] Dobrolecki LE (2016). Patient-derived xenograft (PDX) models in basic and translational breast cancer research. Cancer Metastas. Rev..

[57] Kuperwasser C (2004). Reconstruction of functionally normal and malignant human breast tissues in mice. Proc. Natl Acad. Sci. USA.

[58] Gross S, Piwnica-Worms D (2005). Real-time imaging of ligand-induced IKK activation in intact cells and in living mice. Nat. Methods.

[59] Hu Y, Smyth GK (2009). ELDA: extreme limiting dilution analysis for comparing depleted and enriched populations in stem cell and other assays. J. Immunol. Methods.

[60] Ma CX (2012). Targeting Chk1 in p53-deficient triple-negative breast cancer is therapeutically beneficial in human-in-mouse tumor models. J. Clin. Invest..

[61] Chen X, Chang JT (2017). Planning bioinformatics workflows using an expert system. Bioinformatics.

[62] Callari M (2017). Intersect-then-combine approach: improving the performance of somatic variant calling in whole exome sequencing data using multiple aligners and callers. Genome Med..

[63] Goode DL (2013). A simple consensus approach improves somatic mutation prediction accuracy. Genome Med..

[64] Jia P (2012). Consensus rules in variant detection from next-generation sequencing data. PLoS One.

[65] Li H (2009). The sequence alignment/Map format and SAMtools. Bioinformatics.

