# Supplementary material for: High-resolution clonal mapping of multi-organ metastasis in triple negative breast cancer
Source: Nat Commun. 2018 Nov 29;9:5079. doi: 10.1038/s41467-018-07406-4 (PMC6265294; doi:10.1038/s41467-018-07406-4)
Supplement: Supplementary file 1 — Supplementary Information [file 41467_2018_7406_MOESM1_ESM.pdf]

High-resolution clonal mapping of multi-organ metastasis in triple negative breast cancer.

Echeverria *et al.*

Supplementary Figure 1

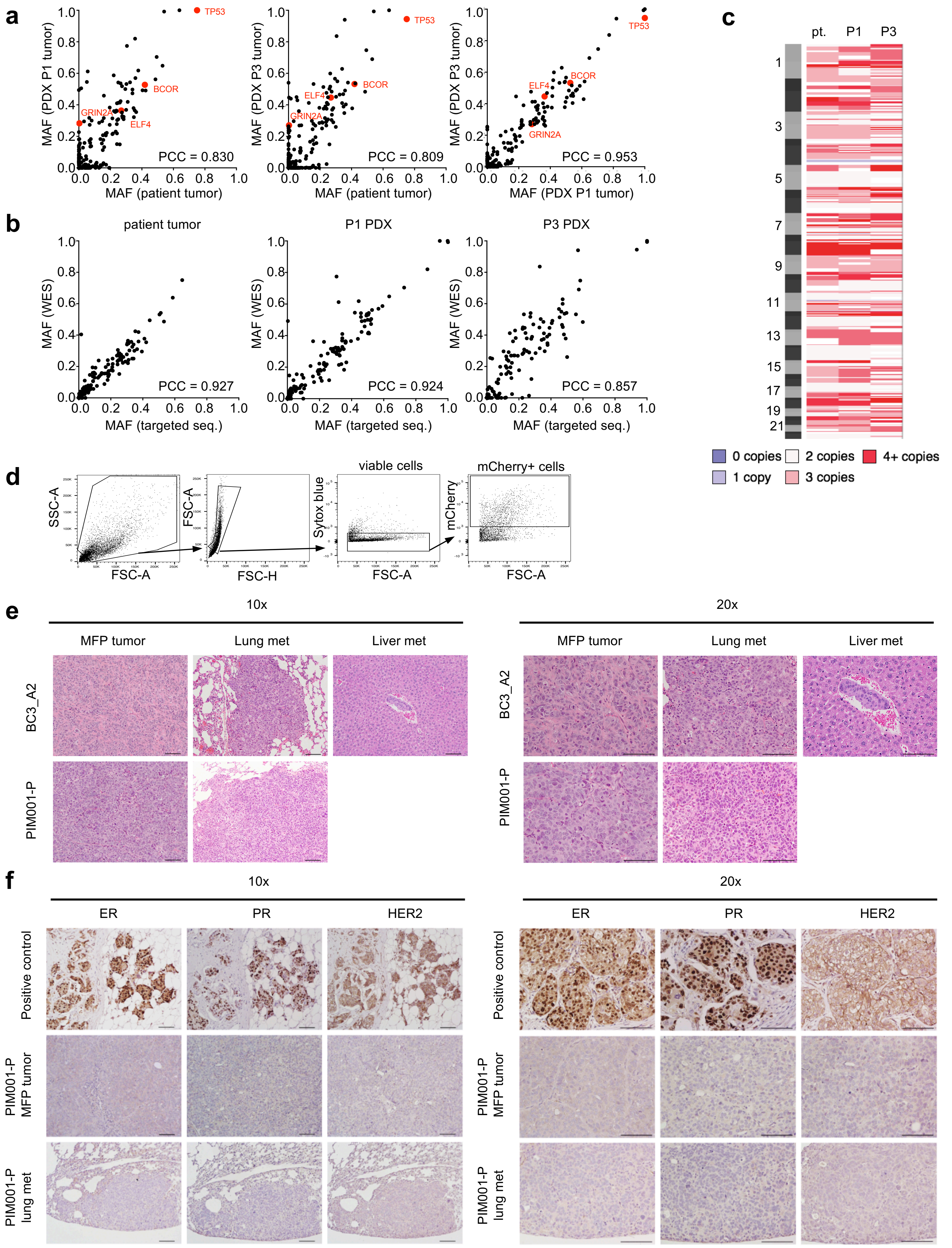

Supplementary Figure 1. Genomic and histologic analyses.

a. MAFs quantified from WES of P1 and P3 tumors were compared with each other and with MAFs of the patient's tumor. Mutations in COSMIC cancer genes are shown in red. Pearson Correlation Coefficients (PCC) are shown in the bottom right corner of each plot.

b. MAFs quantified from WES are compared against those calculated from targeted sequencing in the patient, P1 PDX, and P3 PDX tumors.

c. Exome-wide CN variations in the patient (pt), P1-PDX, and P3-PDX tumors are shown in a heat map. Chromosome numbers are shown on the left. CN values were estimated from the WES data using FACETS.

d. Corresponding to Figure 1e, the gating strategy to sort mCherry+ PIM001-P cells from freshly dissociated PIM001-P tumor cells that had been transduced with the CBRluc/mCherry lentivirus. After sorting, mCherry positive cells were immediately engrafted into MFPs of mice to propagate the PIM1-CBRluc sub-line. Dead cells were excluded by staining with Sytox blue viability dye. FSC – forward scatter, SSC – side scatter, A – area, H – height.

e. H&E staining of MFP tumors and metastases from PDX models used in this study. Scale bars represent 100 μm.

f. Immuno-histochemical staining for Estrogen Receptor (ER), Progesterone Receptor (PR), and HER2 was performed in MFP tumors and lung metastases. A triple-positive human breast tumor sample was used as a positive control for all 3 antibodies. Scale bars represent 100 μm.

Supplementary Figure 2

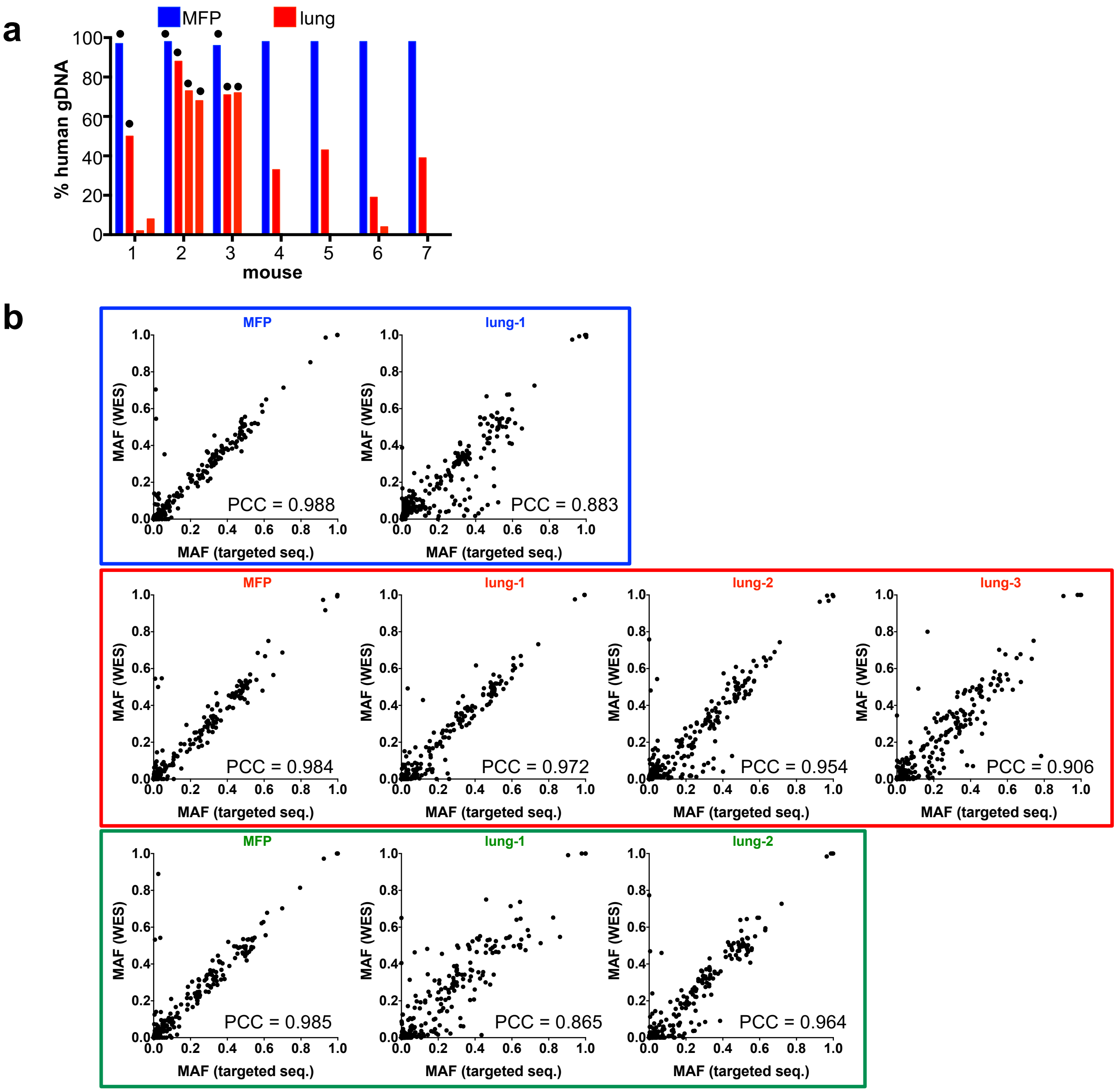

Supplementary Figure 2. Selection and next-generation sequencing of MFP tumors and lung metastases.

a. The percentage of human DNA relative to mouse DNA in MFP tumors and corresponding lung metastases was determined by qPCR. Samples selected for WES are indicated by black dots.

b. Of mutations detected by WES, MAFs quantified from WES are compared against those calculated from targeted sequencing in three replicate MFP tumors and 6 replicate lung metastases. Pearson Correlation Coefficients (PCC) are shown in the bottom right corner of each plot.

Supplementary Figure 3

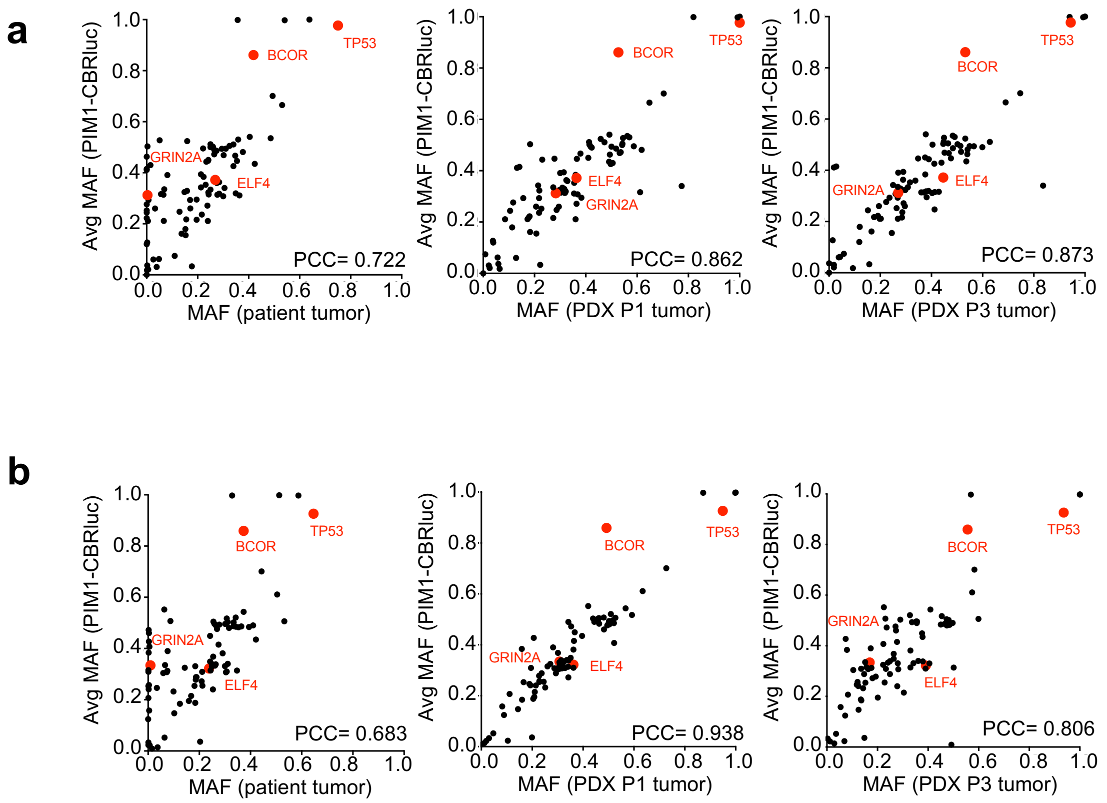

Supplementary Figure 3. Concordance of PIM1-CBRluc with the patient's tumor and early-passage PDX tumors.

a. The average MAFs calculated from WES of PIM1-CBRLuc tumors (n=3) were compared with the patient's tumor, P1 PDX tumor, and P3 PDX tumor by analyzing SNVs detected in both datasets. Pearson Correlation Coefficients (PCC) were calculated (bottom right corner). Mutations in COSMIC cancer genes are shown in red.

b. The average MAFs calculated from targeted sequencing of PIM1-CBRLuc tumors (n=3) were compared with the patient's tumor, P1 PDX tumor, and P3 PDX tumor by analyzing SNVs detected in both datasets. Pearson Correlation Coefficients (PCC) were calculated (bottom right corner). Mutations in COSMIC cancer genes are shown in red.

Supplementary Figure 4

a

MFP	MFP	MFP	Lu-1	Lu-1	Lu-2	Lu-3	Lu-1	Lu-2	
0	31	35	163	77	89	103	91	95	MFP
	0	44	166	82	94	106	96	94	MFP
		0	156	70	84	96	86	84	MFP
			0	122	114	126	122	126	Lu-1
				0	44	60	50	48	Lu-1
					0	60	58	60	Lu-2
						0	64	70	Lu-3
							0	50	Lu-1
								0	Lu-2

b

MFP	MFP	MFP	Lu-1	Lu-1	Lu-2	Lu-3	Lu-1	Lu-2	
1.00	0.96	0.98	0.75	0.79	0.77	0.75	0.76	0.77	MFP
	1.00	0.94	0.75	0.78	0.74	0.75	0.73	0.72	MFP
		1.00	0.77	0.80	0.80	0.77	0.78	0.80	MFP
			1.00	0.93	0.89	0.91	0.88	0.86	Lu-1
				1.00	0.91	0.93	0.91	0.89	Lu-1
					1.00	0.91	0.86	0.86	Lu-2
						1.00	0.89	0.85	Lu-3
							1.00	0.93	Lu-1
								1.00	Lu-2

c

MFP	MFP	MFP	Lu-1	Lu-1	Lu-2	Lu-3	Lu-1	Lu-2	
0	17	31	108	89	98	117	107	114	MFP
	0	42	115	94	103	122	110	119	MFP
		0	99	78	85	104	96	99	MFP
			0	51	34	43	51	44	Lu-1
				0	58	94	58	57	Lu-1
					0	45	45	46	Lu-2
						0	58	57	Lu-3
							0	43	Lu-1
								0	Lu-2

d

MFP	MFP	MFP	Lu-1	Lu-1	Lu-2	Lu-3	Lu-1	Lu-2	
1.00	1.00	0.99	0.68	0.79	0.73	0.71	0.70	0.72	MFP
	1.00	0.98	0.65	0.76	0.70	0.68	0.68	0.69	MFP
		1.00	0.72	0.82	0.76	0.75	0.73	0.75	MFP
			1.00	0.88	0.90	0.88	0.85	0.85	Lu-1
				1.00	0.91	0.90	0.86	0.88	Lu-1
					1.00	0.90	0.83	0.84	Lu-2
						1.00	0.81	0.83	Lu-3
							1.00	0.88	Lu-1
								1.00	Lu-2

e

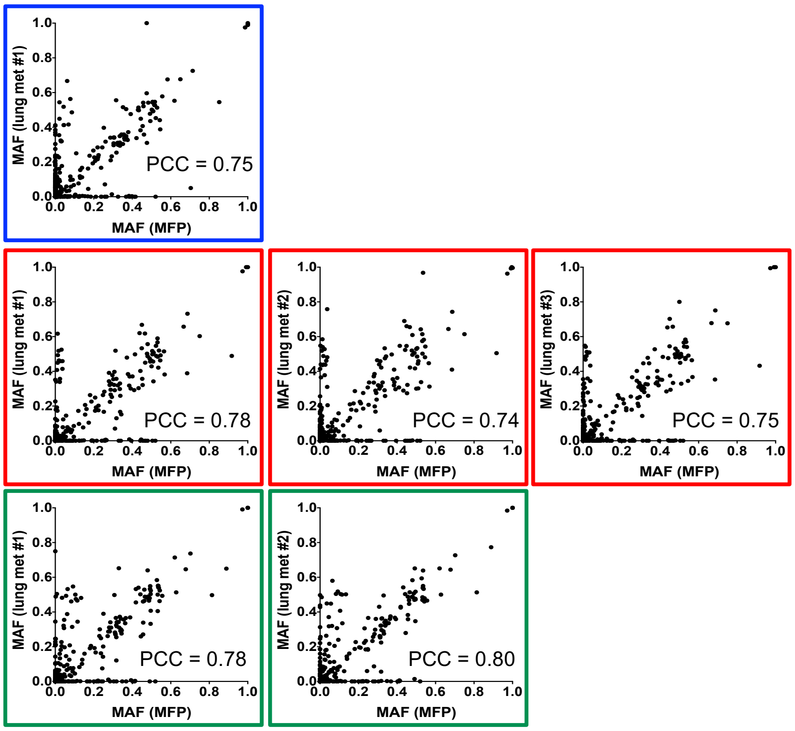

f

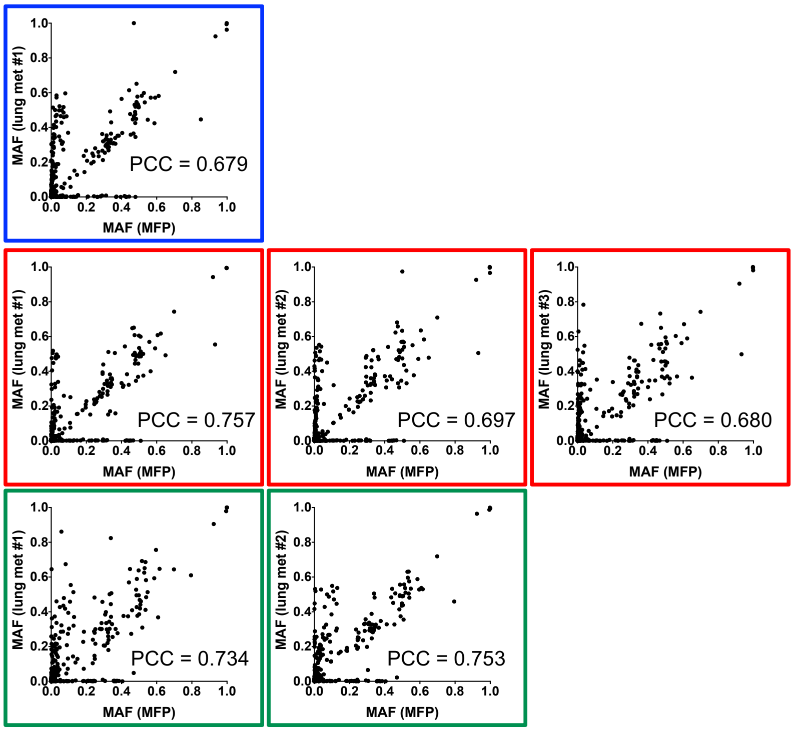

Supplementary Figure 4. Tumor subclones are maintained at different frequencies in primary versus metastatic lesions.

a. Using WES data, pair-wise distance was calculated to compare each MFP tumor and lung metastasis. MFP-MFP comparisons are shaded in blue. Lung metastases compared to their matched MFP tumor are shaded in red.

b. Similarity of MAFs across all MFP tumors and metastases is represented by Pearson correlation coefficients calculated from WES data. MFP-MFP comparisons are shaded in blue. Lung metastases compared to their matched MFP tumor are shaded in red.

c. Using targeted sequencing data of mutations identified by WES, pair-wise distance was calculated to compare each MFP tumor and lung metastasis. MFP-MFP comparisons are shaded in blue. Lung metastases compared to their matched MFP tumor are shaded in red.

d. Similarity of MAFs across all MFP tumors and metastases is represented by Pearson correlation coefficients calculated from targeted sequencing data. MFP-MFP comparisons are shaded in blue. Lung metastases compared to their matched MFP tumor are shaded in red.

e. Using WES data, MAFs of mutations detected in MFP tumors were compared against those in corresponding lung metastases in each mouse (represented as blue, green or red) are shown. Pearson Correlation Coefficients (PCC) were calculated (bottom right corner).

f. Using targeted sequencing data, MAFs of mutations identified by WES detected in MFP tumors were compared against those in corresponding lung metastases in each mouse (represented as blue, green or red) are shown. Pearson Correlation Coefficients (PCC) were calculated (bottom right corner).

Supplementary Figure 5

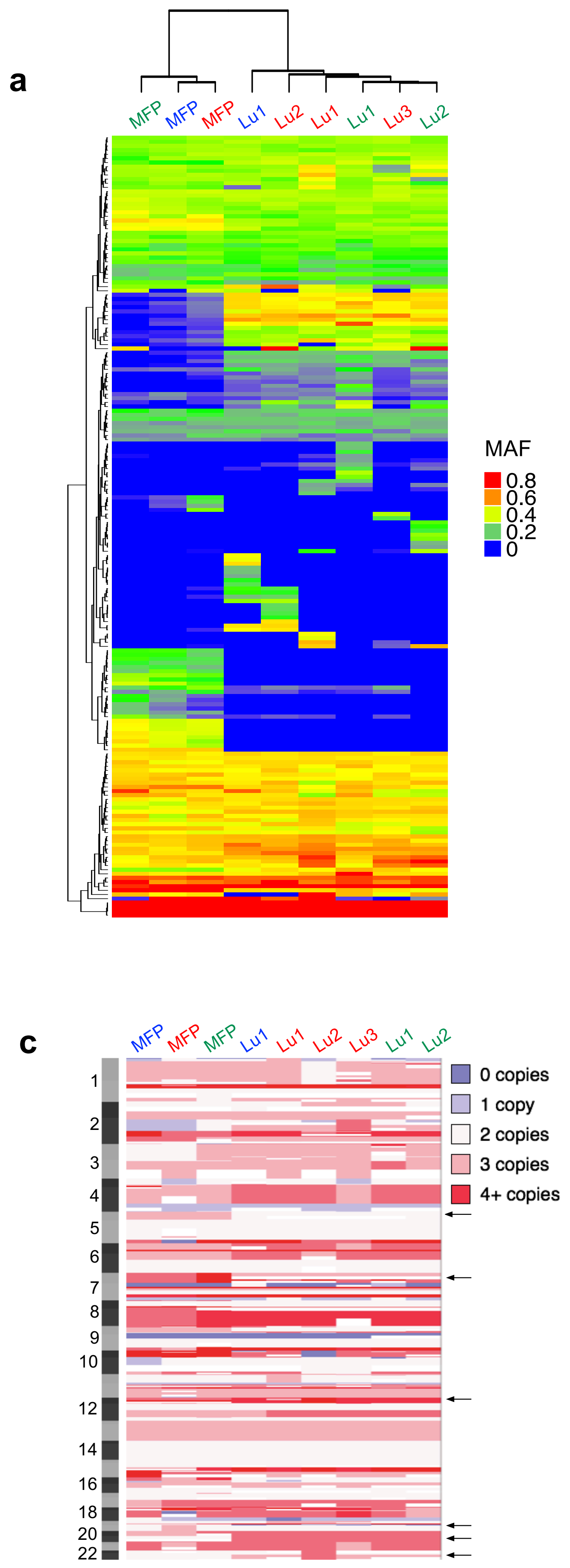

Supplementary Figure 5. Lung metastases exhibit shifts in MAFs and CN status.

a. Non-silent SNVs (MAF$\geq$0.15 in at least one sample) detected by WES are shown in the heat map of MAFs organized by hierarchical clustering.

b. Non-silent SNVs (MAF$\geq$0.15 in at least one sample) detected by WES and validated by targeted sequencing are shown in the heat map of MAFs (calculated from targeted sequencing data) organized by hierarchical clustering.

c. Exome-wide CN alterations detected by WES of MFP tumors and lung metastases are shown in a heat map. Consistent CN alterations in lung metastases are marked with arrows.

Supplementary Figure 6

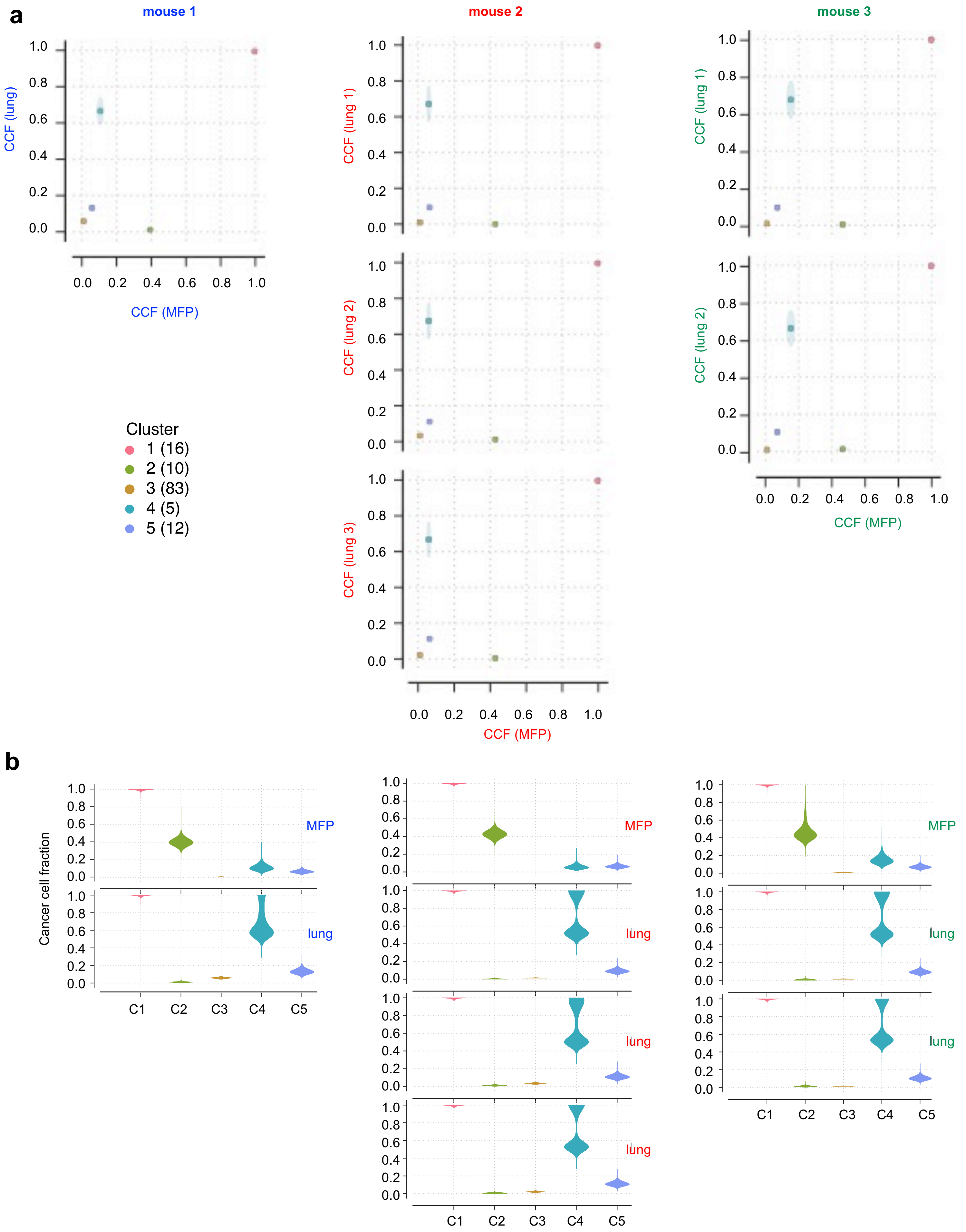

Supplementary Figure 6. PyClone modeling of subclonal architecture.

a. Scatter plots of CCFs (cancer cell frequencies) are shown to compare each lung metastasis to its matched MFP tumor. The mutation clusters are indicated with filled dots, and the shading around each dot represents standard deviation of the estimated CCF. The number of mutations falling within each cluster is shown in parentheses in the legend.

b. The distribution of estimated cellular prevalence for each mutation cluster (C) as calculated by PyClone is shown.

Supplementary Figure 7

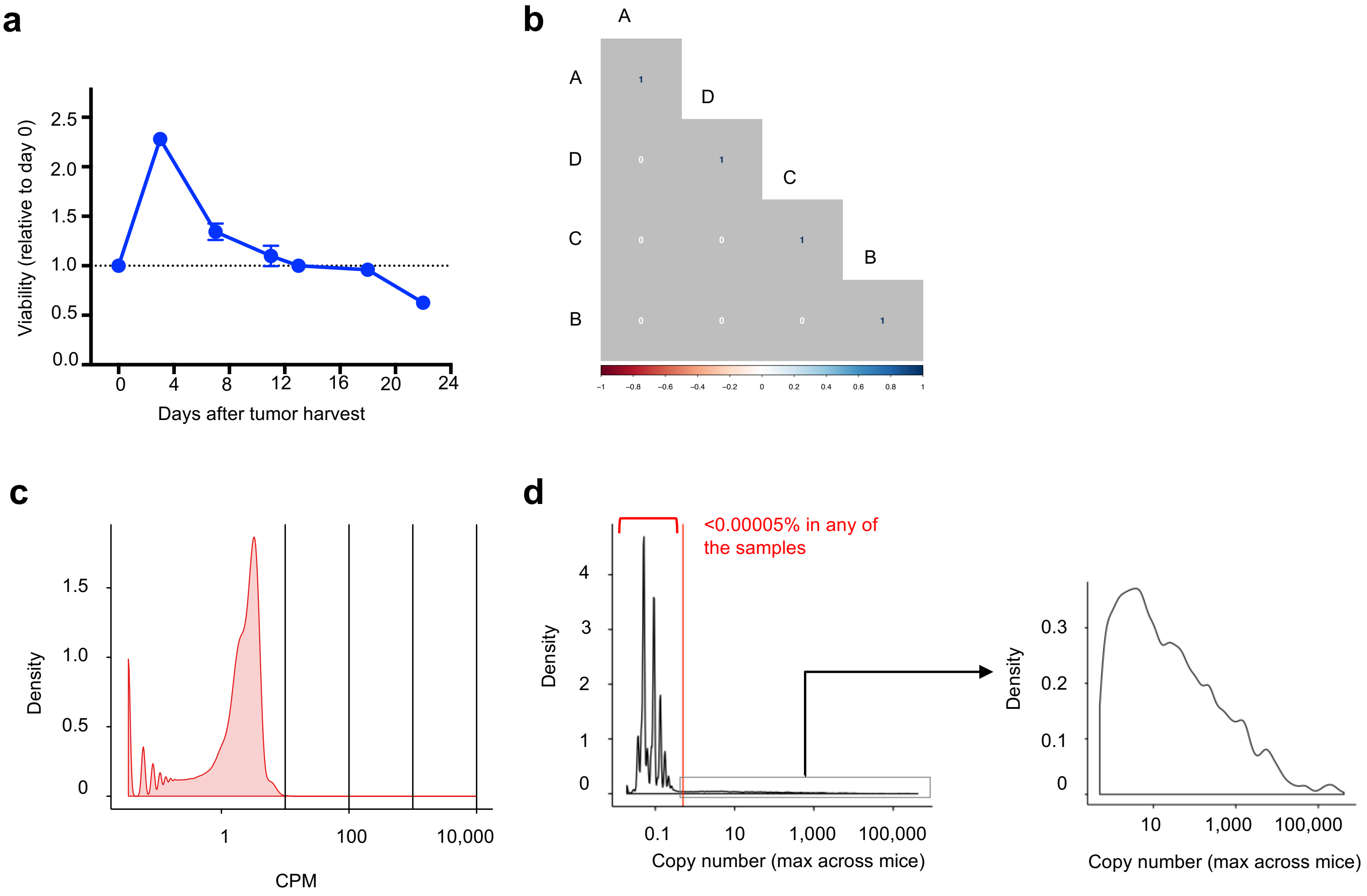

Supplementary Figure 7. High-complexity barcode-mediated clonal tracking

a. Viability of PIM001-P tumor cells was monitored *ex vivo* by Cell-Titer-Glo luminescence assays. Error bars are SEM (n=3 biological replicates).

b. The Pearson correlation coefficients of barcodes detected in the PIM1-CBRLuc MFP tumors of each of the four mice were calculated. Due to the high complexity of the barcode library and low MOI used, each mouse was engrafted with a unique pool of barcodes that did not overlap between mice. Barcodes detected in the top 95% most abundant barcodes in any single lesion across all mice were analyzed.

c. The PIM1-CBRLuc pre-implantation reference cell pellet for the metastasis experiment contained hundreds of thousands of rare unique barcodes, the majority of which had counts of 1-5 CPM.

d. Very rare clones were excluded from barcode analyses because they comprised exceedingly low proportions of the total barcoded population. A density plot of all samples is shown, revealing that many extremely rare barcodes are detected at very low copy numbers. Thus, only barcodes comprising the top 95% of any one sample across all mice were included in analysis of seeding clones.

Supplementary Figure 8

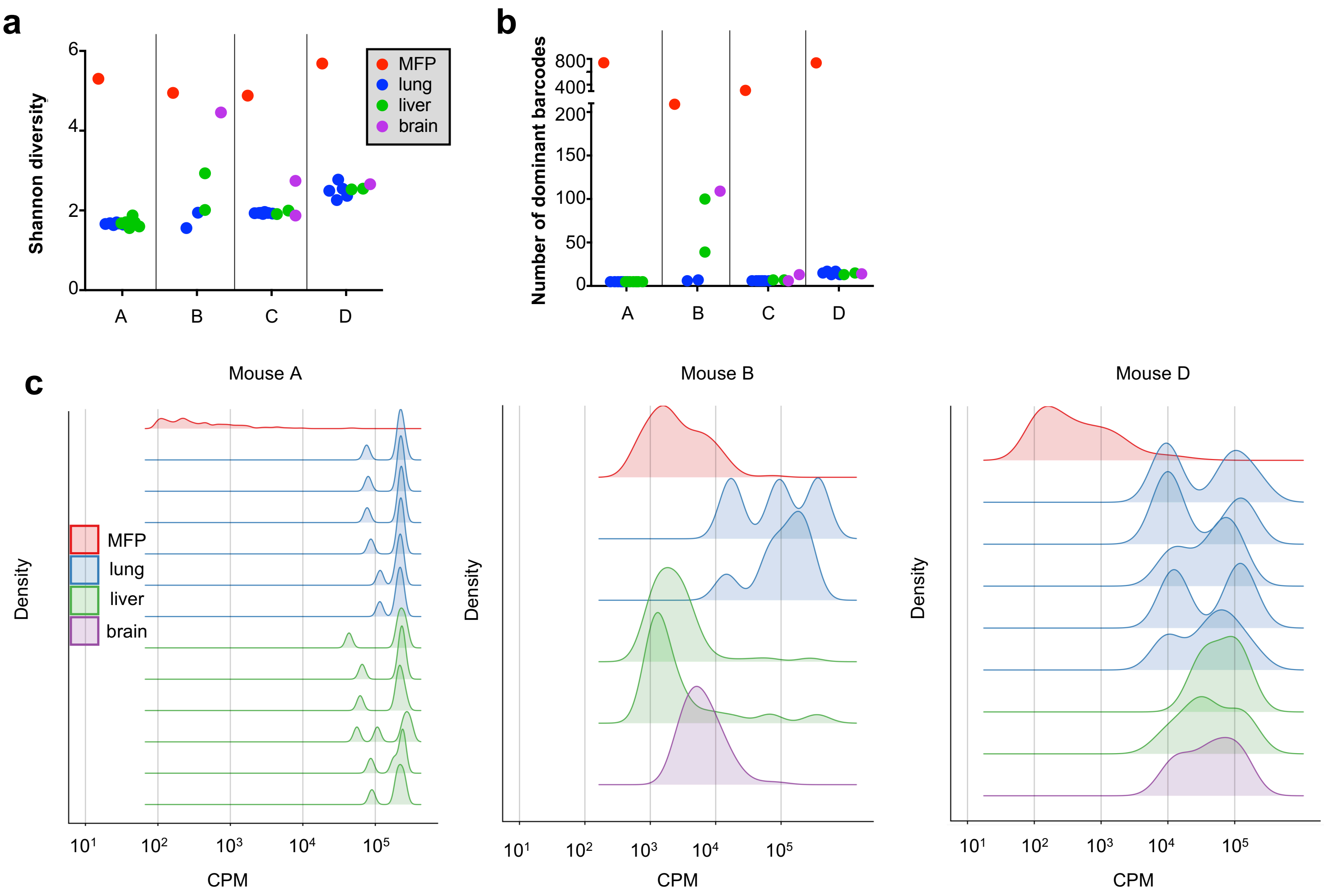

Supplementary Figure 8. Robust alterations in clonal architecture between the primary tumor and corresponding metastases.

a. Shannon Diversity indices were calculated (in nats: natural digits) for all samples in each individual mouse.

b. Numbers of dominant barcodes in each individual tumor sample is shown.

c. The distribution of dominant barcodes (top 95%) in each tumor sample from mice A, B, and D is shown. CPM (counts per million).

Supplementary Figure 9

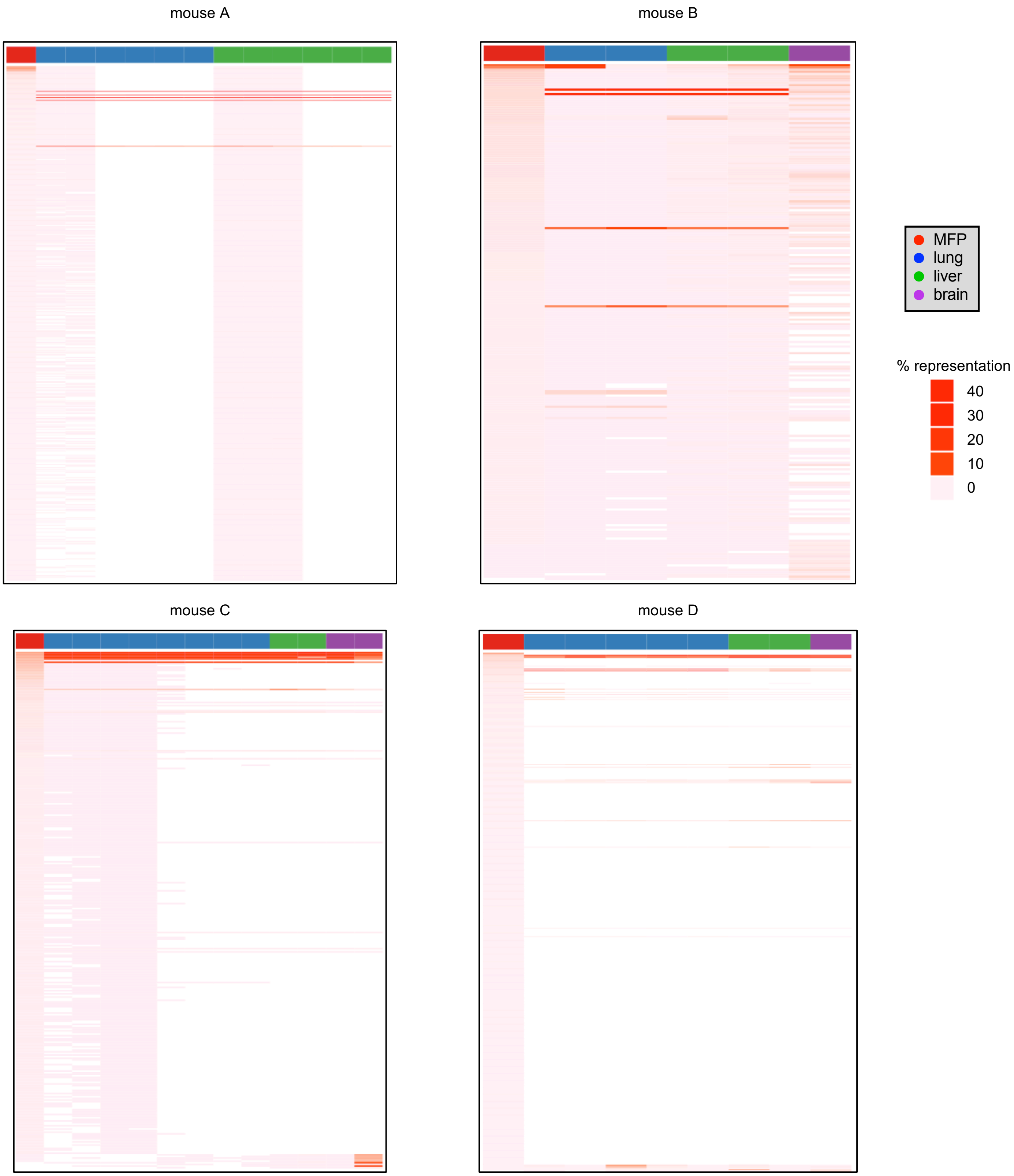

Supplementary Figure 9. Dominant subclones in MFP tumors are not highly represented in corresponding metastases. A heat map of barcode frequencies is shown for each individual mouse. Each heat map includes the top 95% most abundant barcodes in each MFP tumor sample.

Supplementary Figure 10

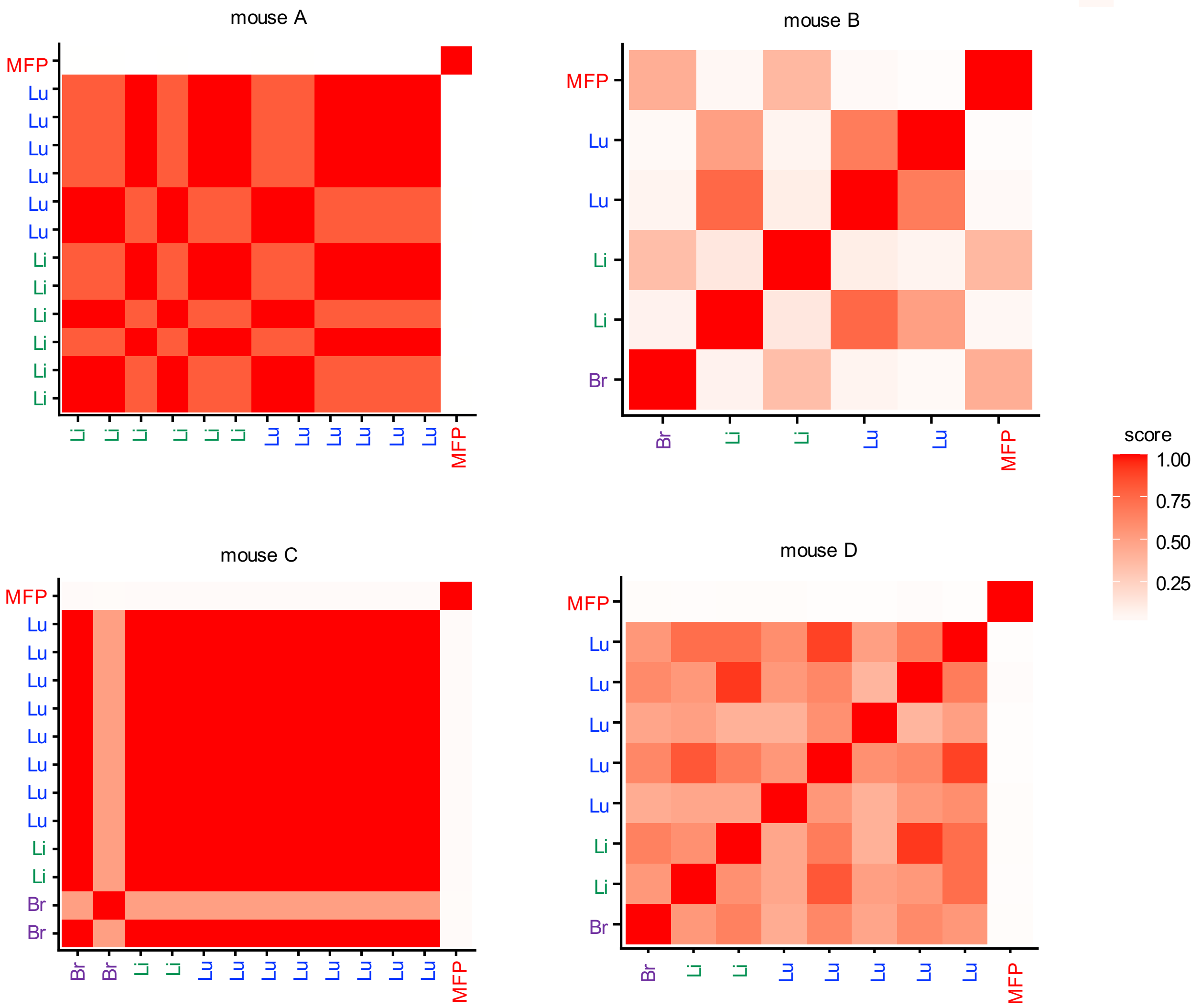

Supplementary Figure 10. Barcode overlap is high between metastases in each mouse.

Jaccard overlap indices were calculated to evaluate the overlap of dominant barcodes (top 95%) between each sample pair within each mouse.

Supplementary Figure 11

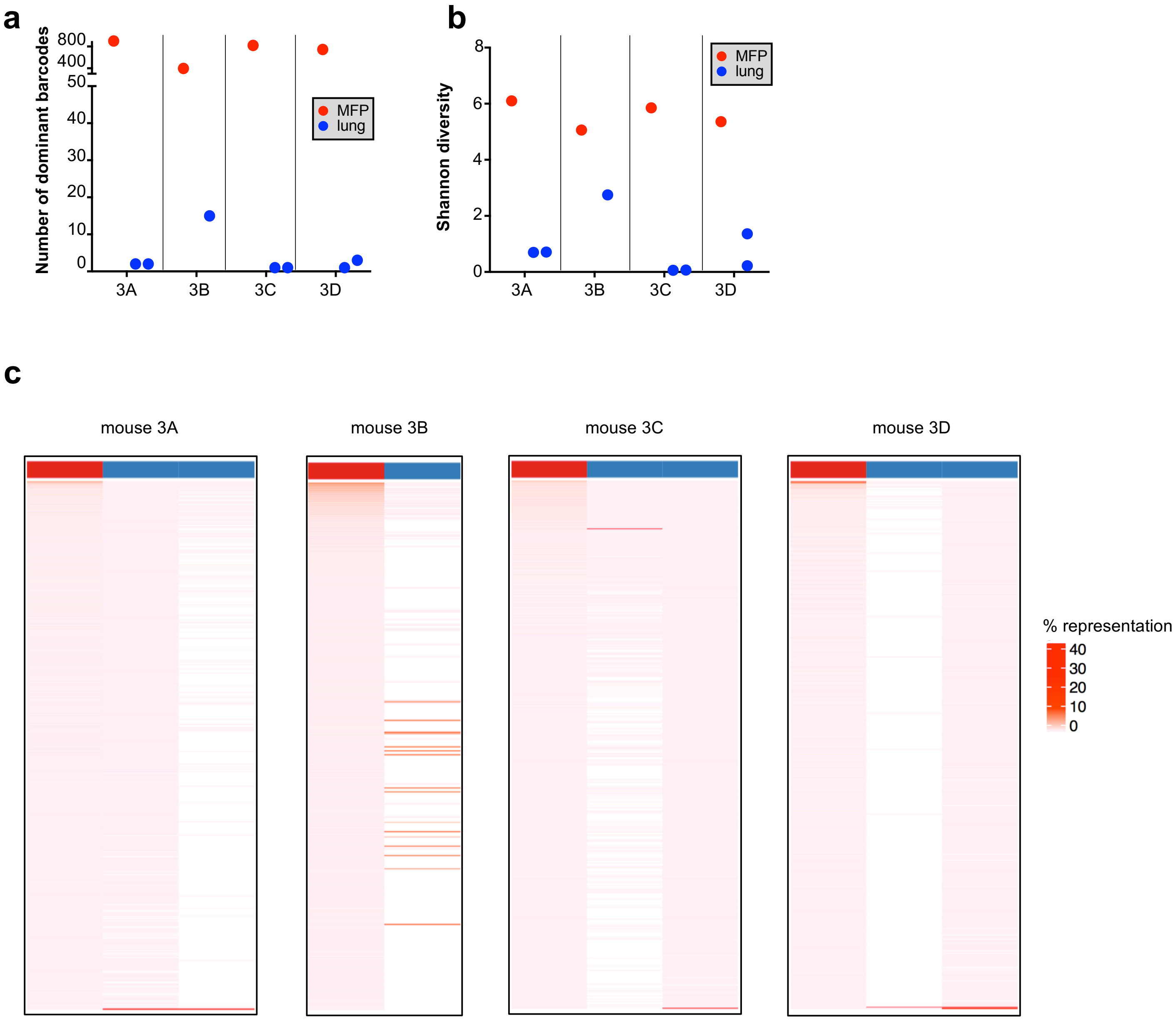

Supplementary Figure 11. Barcode-mediated clonal tracking in BC3_A2.

a. In BC3_A2, unique dominant barcodes (the top 95% most abundant) in each sample were quantified across 4 replicate mice (labeled 3A-3D).

b. In BC3_A2, Shannon Diversity indices were calculated (in nats: natural digits) taking into account all barcodes for each sample as a measure of ITH.

c. In BC3_A2, a heat map of barcode frequencies is shown for each individual mouse. Each heat map includes the top 95% most abundant barcodes in each MFP tumor sample.

Supplementary Figure 12

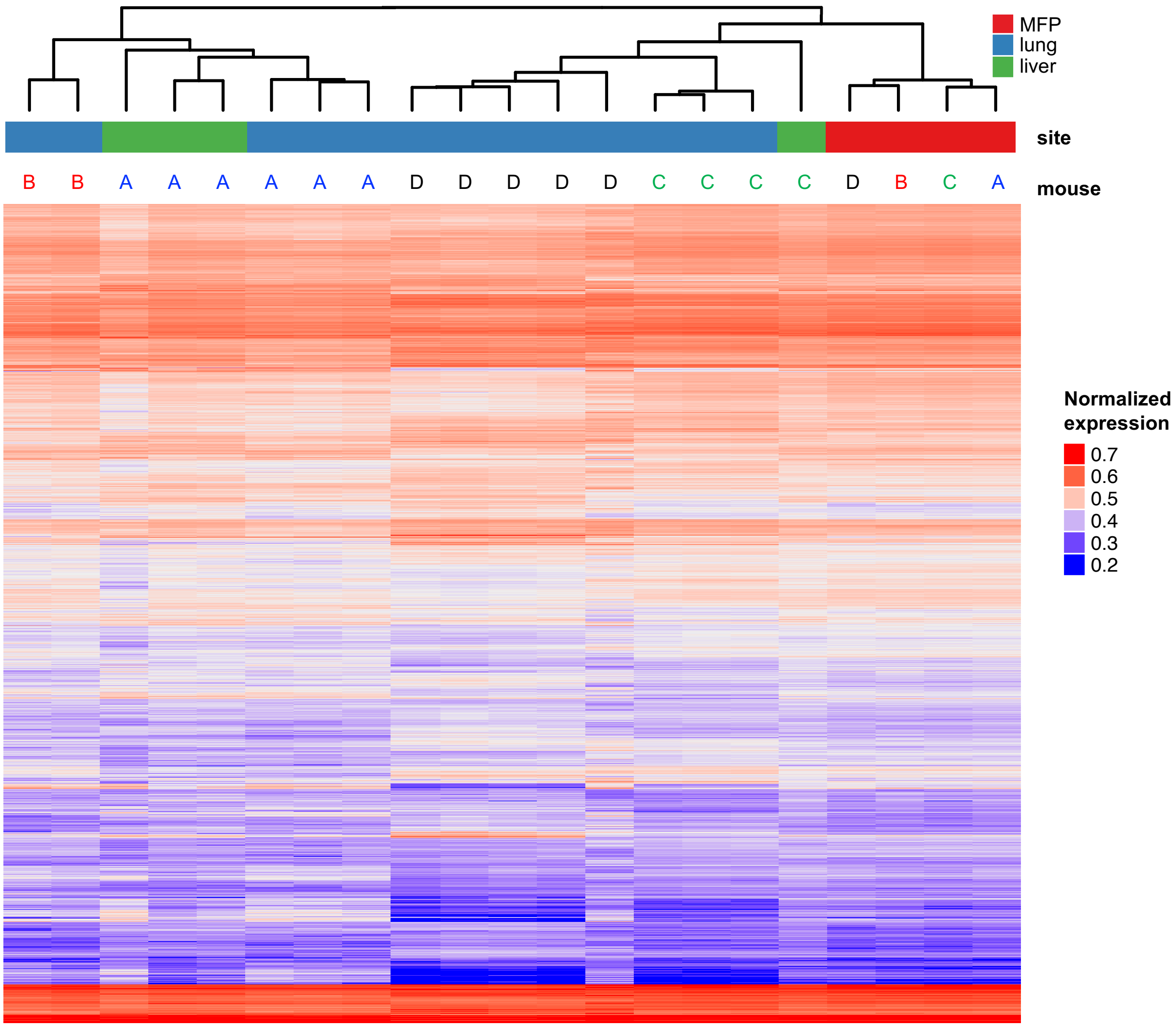

Supplementary Figure 12. Transcriptomic profiling of barcoded MFP tumors, lung metastases, and liver metastases

RNA-seq was conducted, and genes detected in all samples (n=11,450) were used for downstream analysis. These are displayed in a heat map of normalized (size factor normalization implementation in DESeq2) gene expression values organized by unsupervised hierarchical clustering.

Supplementary Figure 13

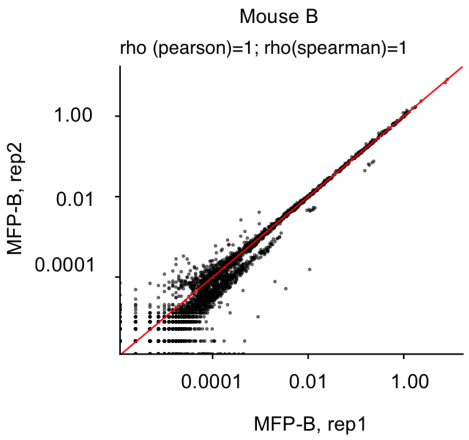

Supplementary Figure 13. Analysis of barcoded technical replicates.

Technical replicate barcode library preparations of mouse B's MFP tumor DNA sample were sequenced and Spearman and Pearson correlations were calculated. Barcode frequencies are plotted on the X and Y axis.
