# Supplementary material for: High-resolution clonal mapping of multi-organ metastasis in triple negative breast cancer
Source: Nat Commun. 2018 Nov 29;9:5079. doi: 10.1038/s41467-018-07406-4 (PMC6265294; doi:10.1038/s41467-018-07406-4)
Supplement: Supplementary file 2 — Description of Additional Supplementary Files [file 41467_2018_7406_MOESM2_ESM.docx]

**Description of Additional Supplementary Files**

**File Name:** Supplementary Data 1.

**Description:** DNA sequencing sample summary. A list of all samples subjected to DNA sequencing (whole-exome sequencing, WES, or targeted DNA sequencing) is shown. The relative percent of human reads (based on alignments to human and mouse reference genomes) is shown. The mean target coverage for each sample is shown.

**File Name**: Supplementary Data 2.

**Description:** Mutations from WES, RNA-seq, and targeted DNA sequencing. Mutation analysis for each individual sample is displayed in a sheet in this workbook. Mutant allele frequencies (MAFs) calculated from WES, RNA-seq, and targeted sequencing are shown.

**File Name**: Supplementary Data 3.

**Description**: Copy number variation segmentation data. Copy number variation (CNV) data for each individual sample is displayed in a sheet in this workbook. Tumor copy number scores generated by FACETS are shown for each segmented region of the genome from WES data.

**File Name**: Supplementary Data 4.

**Description**: Copy number varied genes. Copy number variation (CNV) data for each individual sample is displayed in a sheet in this workbook. Tumor copy number scores generated by FACETS are shown for each gene from WES data.

**File Name**: Supplementary Data 5.

**Description**: RNA-seq data. RNA sequencing data for each individual sample is displayed in a sheet in this workbook. Values are shown in transcripts per million (TPM) for each gene.

**File Name**: Supplementary Data 6.

**Description**: De novo mutation analysis. Putative de novo mutation data is compiled in this workbook. Analyses from WES and targeted DNA sequencing are shown in separate sheets.

**File Name**: Supplementary Data 7.

**Description**: RNA-seq analysis of barcoded samples. RNA sequencing data for each barcoded primary and metastasis sample is displayed in a sheet. Values are shown in transcripts per million (TPM) for each gene.

**File Name**: Supplementary Data 8.

**Description**: Targeted DNA sequencing of barcoded samples. Mutation data generated from analysis of targeted DNA sequencing of each barcoded primary and metastasis sample is shown in a sheet.
